# Phage-prokaryote coexistence strategy mediates microbial community diversity in the intestine and sediment microhabitats of shrimp culture pond ecosystem

**DOI:** 10.3389/fmicb.2022.1011342

**Published:** 2022-09-23

**Authors:** Zhixuan Deng, Shenzheng Zeng, Renjun Zhou, Dongwei Hou, Shicheng Bao, Linyu Zhang, Qilu Hou, Xuanting Li, Shaoping Weng, Jianguo He, Zhijian Huang

**Affiliations:** ^1^State Key Laboratory of Biocontrol, Southern Marine Sciences and Engineering Guangdong Laboratory (Zhuhai), School of Marine Sciences, Sun Yat-sen University, Guangzhou, China; ^2^Maoming Branch, Guangdong Laboratory for Lingnan Modern Agricultural Science and Technology, Maoming, China

**Keywords:** phage, viral community, prokaryotic community, *Litopenaeus vannamei*, shrimp culture pond ecosystem

## Abstract

Emerging evidence supports that the phage-prokaryote interaction drives ecological processes in various environments with different phage life strategies. However, the knowledge of phage-prokaryote interaction in the shrimp culture pond ecosystem (SCPE) is still limited. Here, the viral and prokaryotic community profiles at four culture stages in the intestine of *Litopenaeus vannamei* and cultural sediment microhabitats of SCPE were explored to elucidate the contribution of phage-prokaryote interaction in modulating microbial communities. The results demonstrated that the most abundant viral families in the shrimp intestine and sediment were Microviridae, Circoviridae, Inoviridae, Siphoviridae, Podoviridae, Myoviridae, Parvoviridae, Herelleviridae, Mimiviridae, and Genomoviridae, while phages dominated the viral community. The dominant prokaryotic genera were *Vibrio*, *Formosa*, *Aurantisolimonas,* and *Shewanella* in the shrimp intestine, and *Formosa*, *Aurantisolimonas*, *Algoriphagus,* and *Flavobacterium* in the sediment. The viral and prokaryotic composition of the shrimp intestine and sediment were significantly different at four culture stages, and the phage communities were closely related to the prokaryotic communities. Moreover, the phage-prokaryote interactions can directly or indirectly modulate the microbial community composition and function, including auxiliary metabolic genes and closed toxin genes. The interactional analysis revealed that phages and prokaryotes had diverse coexistence strategies in the shrimp intestine and sediment microhabitats of SCPE. Collectively, our findings characterized the composition of viral communities in the shrimp intestine and cultural sediment and revealed the distinct pattern of phage-prokaryote interaction in modulating microbial community diversity, which expanded our cognization of the phage-prokaryote coexistence strategy in aquatic ecosystems from the microecological perspective and provided theoretical support for microecological prevention and control of shrimp culture health management.

## Introduction

The phage-prokaryote interactions are key topics in microbial community ecology and critical to understanding both microbial diversity and function (Koskella and Meaden, 2013), which can shape the prokaryotes genome, maintain the diversity within or among prokaryotic populations, drive the function of microbial community, and contribute to host health (Diaz-Munoz and Koskella, 2014; Maronek et al., 2020). In the human gut, a multitude of phage-prokaryote interactions occur and shape the gut microbial community (Lim et al., 2015; Dahlman et al., 2021). Phage-prokaryote interactions also play important roles in modulating microbial diversity, potentially impacting honey bee health (Bonilla-Rosso et al., 2020). The transformation of phage-prokaryote interaction improves microbial community adapting to environment-related stress in the heavy metal contamination of soil (Huang D. et al., 2021), cod seeps (Li et al., 2021), and even permafrost (Emerson et al., 2018). The phage-prokaryote interactions are ubiquitous in different environments.

Phage-prokaryote relationships are complex with far-reaching consequences beyond particular pairwise interactions (Diaz-Munoz and Koskella, 2014), on which a lot of researchers have done in-depth research, including the relationship between phage and prokaryotic community diversity (Thingstad and Lignell, 1997; Benmayor et al., 2008), horizontal gene transfer (HGT) between phage and prokaryote (Chen et al., 2021; Zhang et al., 2021), phage survival strategy (Winter et al., 2010; Knowles et al., 2016). Phages are viruses, the most abundant biological entities on earth (Suttle, 2007), which can infect prokaryotes and top-down control microbial abundance by lysis of prokaryotic cells and subsequent release of cellular contents during lytic infection to maintain high biodiversity in marine systems (Breitbart et al., 2018). Phage can also reprogram host metabolism through HGT or auxiliary metabolic genes (AMGs) to adapt the host to environmental changes (Harper et al., 2021; Li et al., 2021). Furthermore, phages have evolved a variety of life history and transmission strategies to reproduce by exploiting prokaryotic host cells. In biological soil crusts, the fastest-growing Firmicutes became primary targets for Caudovirales phages, which were consistent with the Kill-the-Winner (KtW) model (Van Goethem et al., 2019). Similarly, phage and prokaryotic communities fluctuate the KtW model in stable ecosystems and both persist over time in aquatic ecosystems (Rodriguez-Brito et al., 2010). Rohwer and colleagues proposed the Piggyback-the-Winner (PtW) model in an analysis of coral reefs, and lysogeny became increasingly important in high prokaryotic densities ecosystems (Knowles et al., 2016), which was also reflected in the murine gut (Kim and Bae, 2018). These studies suggest that different phage survival strategies could be favored depending on different ecosystem conditions.

Aquaculture has an important role in improving global nutrition and health (Golden et al., 2021). Aquaculture ecosystem has the characteristics of general aquatic system and facilitates the sustainable study of aquatic animals (Zeng et al., 2017; Shekhar et al., 2019). *Litopenaeus vannamei*, also named as the Pacific white shrimp, has the largest production in the global shrimp industry (FAO, 2020). The shrimp culture pond ecosystem (SCPE) is an important anthropogenic aquaculture ecosystem that is continuously fed with additive nutrients during farming (Huang Z. et al., 2021). A large number of studies have been carried out around the shrimp and environment microbiota in SCPE (Zeng et al., 2017; Fan et al., 2019; Gao et al., 2019; Hou et al., 2021), and it has been proved that the shifts and dysbiosis of intestine microbiota and environment microbiota of SCPE are closely associated with shrimp health (Xiong et al., 2015, 2017; Zhu et al., 2016; Huang et al., 2020). In addition, it has been proved that environmental microbial communities shape intestine microbiota for host health in SCPE (Huang Z. et al., 2021). Despite the key role of microbiota in SCPE, the viral communities and phage-prokaryote interaction in the shrimp intestine and cultural sediment are relatively limited studied.

In the present study, we aimed to investigate the relationships of phage-prokaryote and further elucidate their role in the microbial community of the shrimp intestine and cultural sediment. Here, we hypothesize that (i) the prokaryotic and viral communities in shrimp intestine and sediment have different community characteristics at different culture stages; (ii) phage communities are closely related to the prokaryotic communities in shrimp intestine and sediment; and (iii) the phage-prokaryote interactions alter the microbial community composition and function. To test these hypotheses, we collected shrimp intestine and cultural sediment samples at four culture stages during the shrimp culture and analyzed the characteristic of viral communities and prokaryotic communities as well as the phage-prokaryote interaction based on viral metagenomics and 16S rRNA gene amplicon sequencing. Our results will advance understanding of the phage-prokaryote coexistence strategy in aquatic ecosystems from microecological perspective and promote shrimp aquaculture and health management.

## Materials and methods

### Sample collection

The shrimp intestine (named as I) and sediment (named as S) samples of this study were collected in Maoming, Guangdong provinces, China (21.47°N, 111.15°E), from August to October 2020. The shrimp larvae were put into the culture ponds on the 15th day after birth. From the 15th day (stage A) to the 60th day (stage D) after the shrimp larvae were introduced into the ponds, three repeat intestine samples (from 1 to 3) and three repeat sediment samples (from 1 to 3) were collected from three shrimp culture ponds every 15 days (Supplementary Table S1). Sampling was according to the previously reported methods (Hou et al., 2018; Wei et al., 2021). The shrimp’s surface was sterilized with 70% ethanol and the intestine was aseptically dissected. To collect enough samples, shrimp intestines (*n* = 200) were aseptically dissected and mixed into a 2 ml sterile centrifuge tube as one sample. Sediment samples from the depth of 0–3 cm below the surface were collected with core sampler and placed into a 15 ml sterile centrifuge tube. All samples were stored at −80°C until DNA extraction.

### Prokaryotic DNA extraction, sequencing, and data processing

Genomic DNA of shrimp intestine and sediment samples were extracted using ALFA-SEQ Advanced Stool DNA Kit (Magigene, Guangzhou, China) and ALFA-SEQ Advanced Soil DNA Kit (Magigene, Guangzhou, China), respectively. The concentration and purity were measured using the NanoDrop One (Thermo Fisher Scientific, MA, United States). The V4 hypervariable region of 16S rRNA gene was amplified using the primers 515F (5’-GTGCCAGCMGCCGCGGTAA-3′) and 806R (5’-GGACTACHVGGGTWTCTA AT-3′; Bates et al., 2011). Sequencing libraries were generated using NEBNext^®^ Ultra™ II DNA Library Prep Kit (New England Biolabs, MA, United States) following the manufacturer’s recommendations and index codes were added. The library quality was assessed on the Qubit 2.0 Fluorometer (Thermo Fisher Scientific, MA, United States). The library was sequenced on an Illumina Nova6000 platform (Guangdong Magigene Biotechnology Co.Ltd. Guangzhou, China) and 250 bp paired-end reads were generated. The raw data in this study have been deposited in the GenBank Sequence Read Archive database. The accession number is PRJNA796471.

Data quality control and analyses were performed with the DADA2 and quantitative insights into microbial ecology version 2 (QIIME 2; Caporaso et al., 2010). Trimming, merging of paired-end reads, quality filtering, and generating amplicon sequence variants (ASVs) were performed with DADA2 (Kokou et al., 2019). Taxonomic assignments were conducted *via* the ribosome database (Silva.132) (Quast et al., 2013). α-diversity and β-diversity were performed with QIIME 2 (Kusstatscher et al., 2019).

### Virus-like particles enrichment

The shrimp intestine or sediment sample (2 g each sample) was taken to grind and wash with sterile buffer (0.2 M NaCl, 50 mM Tris–HCl, 5 mM CaCl_2_, 5 mM MgCl_2_, pH 7.5), then added ten volumes of the precooled buffer after three rounds of freezing–thawing and removed the cell fragments by 0.22 μm ultrafiltration tube. The samples were centrifuged with centrifuge 3 K30 (Sigma, Osterode, Germany) at 4°C for 5 min at 1000 × *g*, 3,000 × *g*, 5,000 × *g*, 8,000 × *g*, 10,000 × *g*, and 12,000 × *g*, respectively. The cell fragments were removed by a 0.22 μm ultrafiltration tube and the supernatant was transferred to the ultracentrifugation tube containing 28% (w/W) sucrose. After precooling on ice for 10 min, the mixture was centrifuged at 300,000 × *g* for 2 h with HIMAC CP 100wx ultracentrifuge (Hitachi, Tokyo, Japan). After removing the supernatant, the precipitate was suspended in buffer solution (720 μl of water, 90 μl 10× DNase I Buffer, 90 μl 1 U/μl DNase I, 0.9 μl 100 mg/ml RNase A) and shaken at 37°C for 60 min, then stored overnight at 4°C.

### Viral DNA extraction, sequencing, and data processing

Virus DNA was extracted using MiniBEST Viral RNA/DNA Extraction Kit (TaKaRa, Dalian, China), and the whole genome was amplified with REPLI-g Cell WGA & WTA Kit (Qiagen, Hilden, Germany).

Sequencing libraries were generated using NEB Next^®^ Ultra™ DNA Library Prep Kit (New England Biolabs, MA, United States) following the manufacturer’s recommendations and index codes were added. The library quality was assessed using the Qubit^®^ dsDNA HS Assay Kit (Life Technologies, Grand Island, NY) and Agilent 4200 system (Agilent, Santa Clara, CA). The libraries were sequenced on an Illumina NovaSeq 6000 and 150 bp paired-end reads were generated. The raw data in this study have been deposited in the GenBank Sequence Read Archive database. The accession number is PRJNA797730.

The raw data processing with SOAPnuke (v1.5.6; Chen et al., 2018) were conducted to acquire the clean data for subsequent analysis. To remove the host sequence, clean reads were compared with the ribosome database (Silva.132) and host reference genome of Pacific white shrimp (NCBI Project ID: PRJNA438564; Zhang et al., 2019), respectively, with Burrows-Wheeler Aligner (BWA) software (v0.7.17, parameter: mem –k 30; Li and Durbin, 2009). The results that comparison length was less than 80% of the length of reads would be filtered, and then removed the host sequence (Li and Durbin, 2009).

### Viral contigs assembly, identification, functional gene annotation, and host prediction

Clean data were assembled with Megahit software (v1.1.2, parameter: -presets meta-large-min-contig-len 300; Li et al., 2016). To calculate the utilization ratio of reads, clean reads were compared with assembly results by BWA software (v0.7.17) (Li and Durbin, 2009). To remove the host sequence, contigs were compared with the host reference genome of Pacific white shrimp by BLAST software (v2.9.0+; Camacho et al., 2009). The contigs of all samples were clustered to obtain unique contig with CD-HIT software (v4.7, parameter: -c 0.95 -aS 0.8; Li and Godzik, 2006).

The unique contigs were compared with virus database separated from nucleotide (NT) sequence database with BLAST software (v2.9.0+; Camacho et al., 2009). The virus sequence was defined with alignment similarity ≥ 80%, the alignment length ≥ 500 bp, and e ≤ 1e-5. The suspected virus sequence was defined with the alignment length ≥ 100 bp, and e ≤ 1e-5. The contigs were further compared with virus database separating from non-redundant protein sequence database (Pruitt et al., 2005) and hidden Markov models (viral protein families and viral profile; Rabiner and Juang, 1986), and the union as the candidate virus sequences was obtained. The candidate virus sequences were compared with NCBI taxonomy database (Federhen, 2012). If more than 20% of the first 50 alignment results supported the sequences which were non-viral sequences, the candidate virus sequences would be excluded and the rest would be considered as new virus sequences (Li et al., 2008; Li and Durbin, 2009; Paez-Espino et al., 2016; Zhao et al., 2017; El-Gebali et al., 2019).

According to the comparison results between the virus contigs and the virus NT database, the best hit with e < 1e-5 was selected for species annotation. The clean reads were compared with virus contigs using BWA software (v0.7.17, parameter: mem -k 30; Li and Durbin, 2009), then the results that comparison length was less than 80% of the length of reads would be filtered. The relative abundances of virus contigs were expressed by reads per kilobase per million mapped reads (RPKM) values (Wagner et al., 2012).

The gene sequence of the virus contigs was predicted and the predicted gene number and length were evaluated with MetaGeneMark (v3.38; Zhu et al., 2010). The gene sequence was compared with the virus sequence of the UniProtKB/ SWISS-PROT database (ViralZone)^1^ with BLASTp software (v2.9.0+; Lavigne et al., 2008), and the best hit comparison result with e < 1e-3 was screened to obtain the virus function information.

Construction of the CRISPR-Cas spacer database was from bacterial genome of the RefSeq database with CRISPR recognition tool (CRT)^2^ (Bland et al., 2007). The identified viruses were compared with CRISPR-Cas spacer database with blastn-short (v2.9.0+; Camacho et al., 2009). The best hit was selected as the possible host information of phage when the e-value was < 1e-10, the comparison similarity was more than 95%, and the coverage of spacer was more than 80%.

### Co-occurrence network construction

Co-occurrence network analysis (Coutinho et al., 2017) was performed on phage and prokaryote of shrimp intestine and sediment using the “igraph” and “psych” libraries with R and Gephi software (Bastian et al., 2009). To reduce complexity, only ASVs (or genus) and contigs occurring in more than 50% of all samples were retained. The pairwise Spearman’s correlations between those were calculated, with a correlation coefficient > |0.8| (or > |0.5|) and a *p* < 0.05 being considered as a valid relationship. Procrustes analysis (Goodall, 1991) was performed based on the Bray-Curtis distances of eigenvalues for both prokaryote and phage with Vegan packages (Cao et al., 2021).

### Statistical analysis

Venn diagrams were constructed with the R package VennDiagram (Chen and Boutros, 2011). The Shannon index and richness of prokaryote and viral communities were estimated with the R package Vegan (Oksanen et al., 2007). The composition differences of different samples were compared through β-diversity analysis, and the non-metric multidimensional scaling (NMDS) was obtained according to the Bray-Curtis distance matrix (Vandeputte et al., 2017).

The biomarkers of each culture stage based on ASVs (prokaryote) and contigs (virus) of sequence were searched with linear discriminant analysis Effect Size (LEfSe) analysis. Prokaryote ASVs and virus contigs with significant differences in abundance among different stages were detected with nonparametric Kruskal-Wallis test (K-W test; Kruskal and Wallis, 1952). The difference between the two culture stages was judged with Wilcoxon rank-sum test. The impact of significant virus and prokaryote was evaluated with linear discriminant analysis (LDA) (Vandeputte et al., 2017). The difference in the α-diversity values consequences between the compared groups was tested by K-W test with SPSS (Version 21). Analysis of similarities (ANOSIM) was used to assess and validate whether the groups showed significant differences in virus or prokaryote community composition with the default 999 permutations using R package Vegan (Cao et al., 2021). The relationships between the α-diversity and Bray-Curtis distance of the virus and prokaryote, and the relationships between the abundance of virus contigs and their predicted host were determined with Spearman rank correlation. The *p*-values for multiple comparisons were adjusted with the false discovery rate (Benjamini and Hochberg, 1995).

## Results

### The prokaryotes communities in shrimp intestine and sediment were different at four culture stages

**Figure 1:**
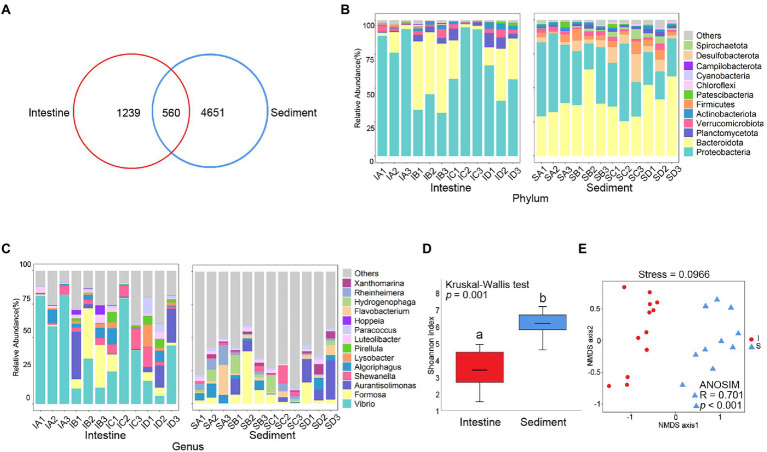
Comparative analysis of prokaryotic community between shrimp intestine and sediment. **(A)** Venn analysis of prokaryotic communities at the amplicon sequence variant (ASV) level among shrimp intestine (red) and sediment (blue). Relative abundances of **(B)** dominant prokaryotic phyla and **(C)** dominant prokaryotic genera. **(D)** Box plots figure showed the range of Shannon index in shrimp intestine (red) and sediment (blue). The significant difference calculated by the Kruskal-Wallis test was shown by marking a and b. **(E)** The β-diversity of prokaryotic communities of shrimp intestine (red) and sediment (blue) was analyzed by NMDS and ANOSIM based on the Bray-Curtis distance.

A total of 6,450 prokaryotic ASVs were detected from 24 samples. Venn diagram analysis showed that there were 560 shared prokaryotic ASVs between shrimp intestine and sediment (Figure 1A), 109 shared prokaryotic ASVs among four culture stages in shrimp intestine (Supplementary Figure S1A) and 258 shared prokaryotic ASVs among four culture stages in sediment (Supplementary Figure S1B).

Prokaryote of the Proteobacteria and Bacteroidetes phylum were the most abundant prokaryotic taxa, which were identified both in shrimp intestine and sediment (Figure 1B). At genus level, the majority belonged to *Vibrio*, *Formosa*, *Aurantisolimonas,* and *Shewanella* were detectable in shrimp intestine, while the majority belonged to *Formosa*, *Aurantisolimonas*, *Algoriphagus,* and *Flavobacterium* were detectable in sediment (Figure 1C). The Shannon index of shrimp intestine was significantly lower than those in sediment (*p* < 0.001; Figure 1D; Supplementary Table S2), but there was no significant (*p* > 0.05) difference among the four culture stages (Supplementary Figure S1C; Supplementary Table S2). The overall prokaryote composition was distinct among samples in shrimp intestine (*p* < 0.001) and sediment (*p* < 0.001; Figure 1E). In addition, the prokaryotic composition of the shrimp intestine (*p* < 0.01; Supplementary Figure S1D) and sediment (*p* < 0.001; Supplementary Figure S1E) were significantly different at four culture stages.

The LEfSe analysis was used to identify the specific prokaryote phylotypes that were differentially altered among the four culture stages (Supplementary Figures S1F,G). The discriminative features of ASV11 (LDA = 4.63, *p* = 0.040) and ASV82 (LDA = 3.66, *p* = 0.025) were detected, which were both belong to *Vibrio* and were significantly higher in shrimp intestine at the Stage C (Supplementary Table S3).

### The communities of virus in shrimp intestine and sediment were different at four culture stages

**Figure 2:**
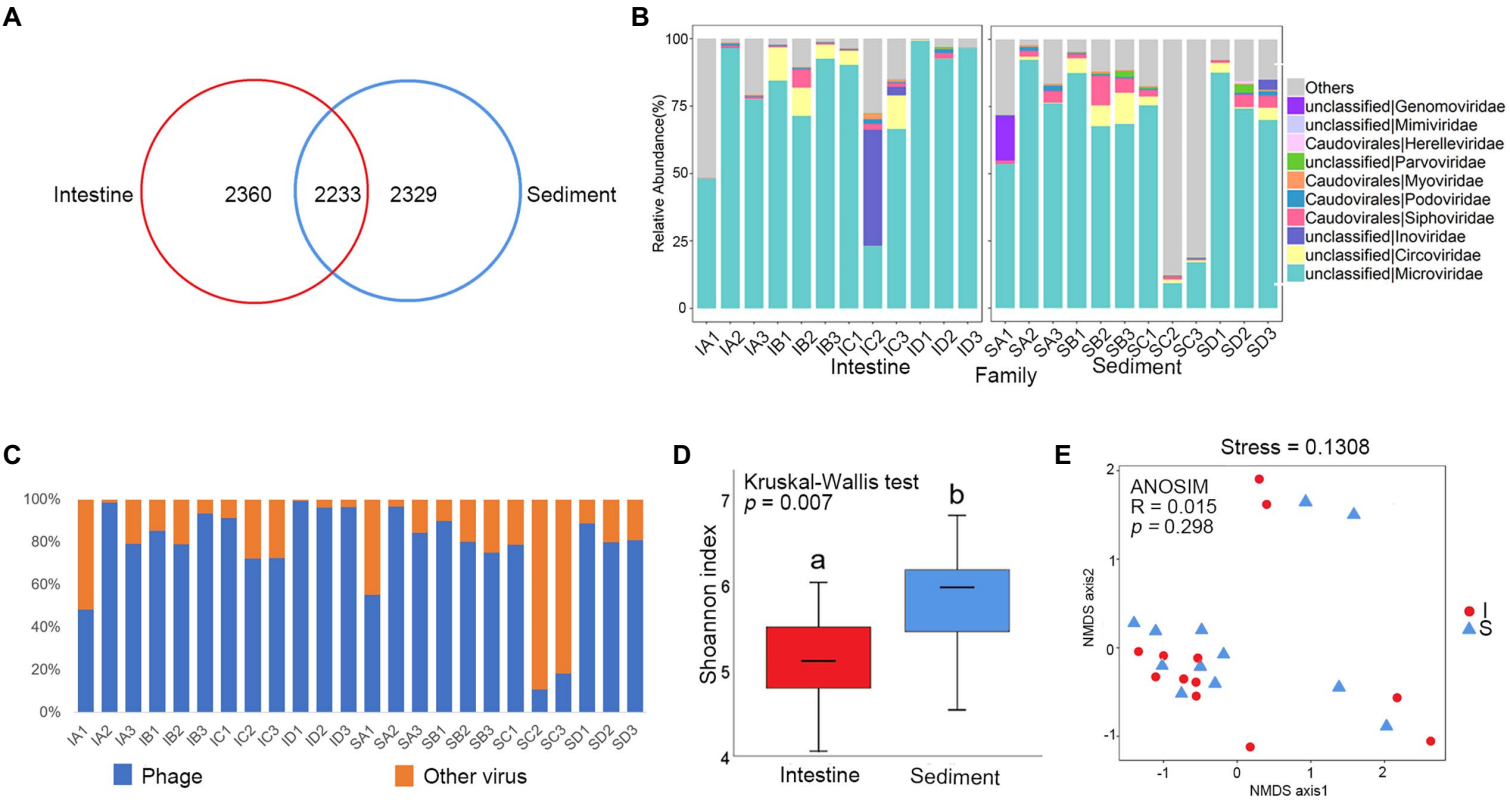
Comparative analysis of viral community between shrimp intestine and sediment. **(A)** Venn analysis of viral communities at the contig level among shrimp intestine (red) and sediment (blue). **(B)** Relative abundances of dominant viral families. **(C)** Relative abundances of phage and other viruses. **(D)** Box plots figure showed the range of Shannon index in shrimp intestine (red) and sediment (blue). The significant difference calculated by the Kruskal-Wallis test was shown by marking a and b. **(E)** The β-diversity of viral communities of shrimp intestine (red) and sediment (blue) was analyzed by NMDS and ANOSIM based on the Bray-Curtis distance.

A total of 6,622 contigs were obtained by assembling the reads. Venn diagram analysis checked the shared viral contigs between shrimp intestine and sediment, and among four culture stages. There were 2,233 shared contigs between intestine and sediment (Figure 2A), and 575 and 681 shared contigs among different culture stages in shrimp intestine (Supplementary Figure S3A) and sediment, respectively (Supplementary Figure S3B).

In order to characterize the taxonomic composition of the virus, all 6,622 dereplicated and well-covered contigs that exploited the information in both the assembled contigs and their encoding proteins were annotated. The viral sequences could be assigned to 28 different viral families. The majority of the viral contigs both in shrimp intestine and sediment were assigned to DNA viral families, including Microviridae, Circoviridae, Inoviridae, Siphoviridae, Podoviridae, Myoviridae, Parvoviridae, Herelleviridae, Mimiviridae and Genomoviridae (Figure 2B). Phages were the most commonly detected viruses in most of the samples, except for IA1, SC2, and SC3 (Figure 2C). Phages from families Microviridae, Siphoviridae, Podoviridae, Myoviridae, and Herelleviridae were the most frequently found viruses both in shrimp intestine and sediment. The relative abundance of Microviridae was average 78 and 65% of the total viral sequences in the intestine and sediment, respectively. In IC2 from the intestine, more than 43% of the total viruses were composed of Inoviridae. This result revealed that an unclassified taxonomic fraction of contigs (around 18%) were without any assignment to a known viral taxon after BLAST analysis. Sequences were also assigned to many less-abundant viral taxa including phages, whose annotated hosts included prokaryotes commonly found in *L. vannamei* intestine and common eukaryotic viruses (Figure 2B; Supplementary Figure S2A).

α-Diversity was significantly lower in the shrimp intestine compared with in sediment (*p* < 0.01; Figure 2D), while there was no significantly different between the four culture stages both in shrimp intestine and sediment (Supplementary Figure S3C). There was no significantly different in viral composition between shrimp intestine and sediment (*p* > 0.05; Figure 2E), but significantly different among four culture stages in shrimp intestine (*p* < 0.05; Supplementary Figure S3E) and in sediment (*p* < 0.01; Supplementary Figure S3F).

The LEfSe analysis was used to identify the specific phage phylotypes that were different alterations among the four culture stages (Supplementary Figures S3G,H). The following discriminative features were detected: IB1 contig 17,174 (LDA = 4.234, *p* = 0.036), IC1 contig 7,070 (LDA = 4.250, *p* = 0.034), IB3 contig 10,716 (LDA = 4.237, *p* = 0.034), and IA1 contig 5,874 (LDA = 4.245, *p* = 0.034), which were both annotated as *Vibrio* phage and were significantly higher in shrimp intestine at the stage C (Supplementary Table S3).

The majority of contigs from metagenomes mapped to genes with unknown function. Along with phage-related functions, phage contigs were also annotated as genetic information processing genes and diverse AMGs (Supplementary Figure S4), such as nucleotide metabolism (137 genes), the metabolism of cofactors and vitamins (77 genes), and amino acid metabolism (63 genes) and energy metabolism (9 genes) (Supplementary Table S5). In addition, some phages contig with toxin genes, such as zona occludens toxin and putative accessory cholera enterotoxin, were annotated as Inoviridae. These general pathway results indicated that phages presumably altered host metabolism and viability through the consumption of metabolic resources needed for their own biogenesis.

### The phage communities were closely related to the prokaryotic communities

**Figure 3:**
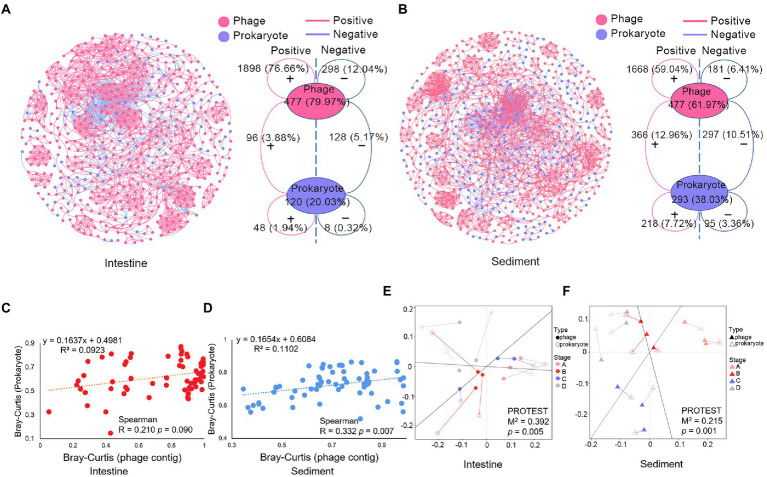
Correlations analysis of the microbial community in shrimp intestine and sediment. Co-occurrence network of **(A)** shrimp intestine microbial community, **(B)** sediment microbial community, and topological information of the microbial community co-occurrence network. The positive or negative linkages of the association networks were based on positive or negative Spearman’s correlations between any pairs of nodes. Phage contigs (red) and prokaryotic ASVs (blue) are network nodes, the red line means positive correlations, and the blue line means negative correlations. Plot showed the relationship between the Bray-Curtis distance of phage (contigs level) and prokaryotic (ASV level) community in **(C)** shrimp intestine and **(D)** sediment, and the Spearman correlation coefficient is indicated. Procrustes analysis depicting the correlation between phage community (solid graphics) and prokaryotic community (hollow graphics) on the basis of Bray–Curtis dissimilarity metrics in **(E)** shrimp intestine and **(F)** sediment.

Co-occurrence network based on Spearman’s correlations was constructed to analysis complex relationships between phages and prokaryotes. In shrimp intestine, most of the links in the network were intra-phage associations (88.7% of a total of 2,476 links). In addition, there were 9.05% phage-prokaryote links and 2.26% prokaryote-prokaryote links (Figure 3A). Similarly, most of the links in the network were intra-phage associations in sediment (65.45% of a total of 2,825 links) (Figure 3B). Moreover, there were 23.47% phage-prokaryote links and 11.08% prokaryote-prokaryote links in sediment which were more than that in shrimp intestine. Most of the phage-phage pairs (86.43% and 90.21% in intestine and sediment, respectively) were positively linked (Figures 3A,B), suggesting a possibility of coinfection or co-habitat. The number of positive and negative links of phage-prokaryote pairs in the shrimp intestine and sediment were almost the same.

The α-diversity and β-diversity of the virus were examined using contigs and species two different layers of information which were recovered from the sequence data. The α-diversity of the virus and the prokaryote were negatively correlated only in the species layer, but not in the contig layer (Supplementary Table S6). The Shannon index of viruses was negatively correlated with those of prokaryotes in shrimp intestine and with the richness of prokaryotes in sediment (Supplementary Table S6). Furthermore, the Shannon index of phage and prokaryote was tended to be negatively correlated, but not significantly (*p* > 0.05; Supplementary Table S6). The pairwise distance matrices showed a positive correlation between β-diversity measures of phage and prokaryote in sediment (*p* < 0.05) but not in shrimp intestine (*p* > 0.05) which was based on contig and species layers of information (Figures 3C,D; Supplementary Figures S5A,B). Moreover, Procrustes analysis revealed that significant associations between the community of phage and prokaryote were both in shrimp intestine (*M*^2^ = 0.392, *p* = 0.005; Figure 3E) and sediment (*M*^2^ = 0.215, *p* = 0.001; Figure 3F). All these data indicated that the phage communities were closely related to the prokaryotic communities.

### Phage-host interactions

**Figure 4:**
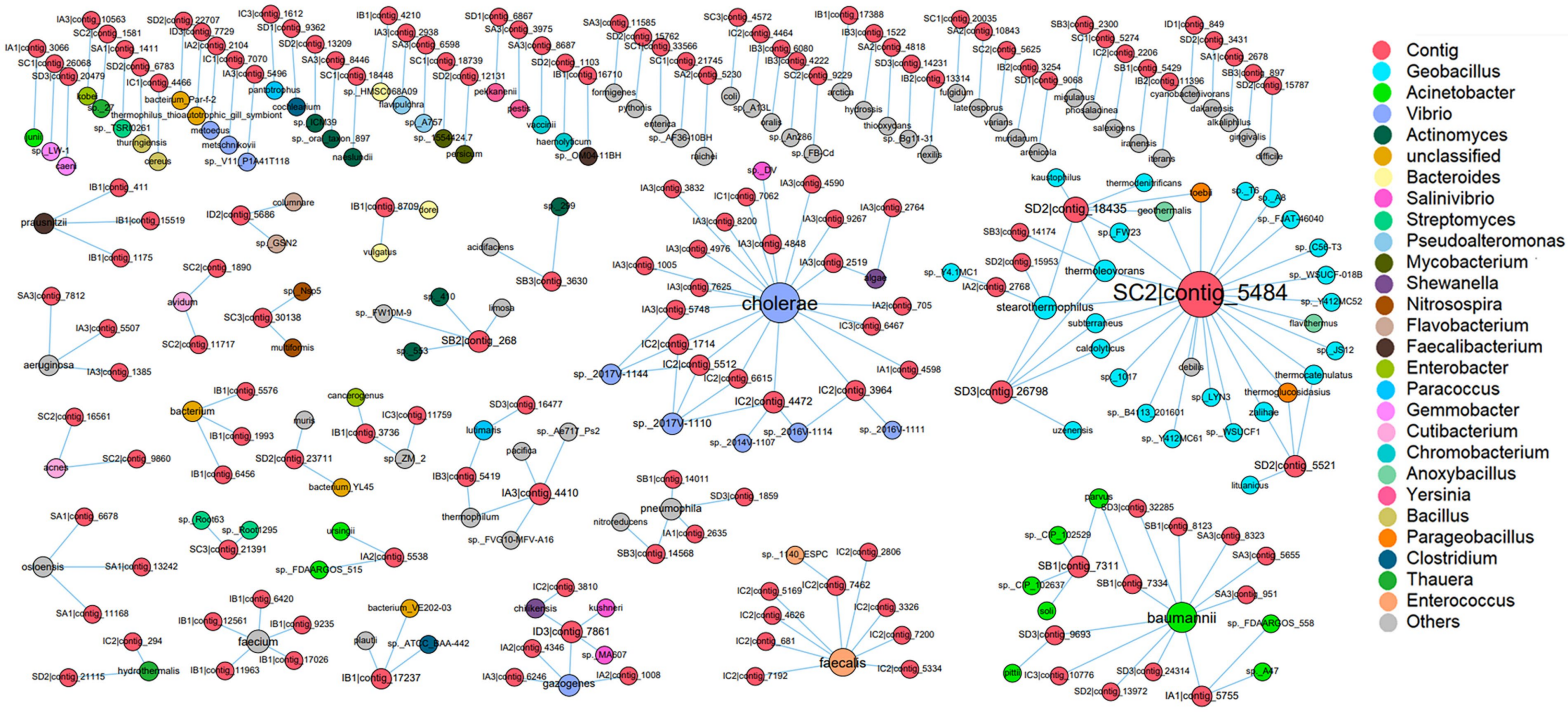
The host prediction of virus. Within the network, viruses (contig level) are represented as red nodes, and prokaryotes (species level) are represented as other color nodes, each node color indicates a prokaryotic genus, and the relative size of nodes is proportional to the number of interactions between virus and prokaryote taxa identified. The edge between a virus node and a prokaryote node indicates their infection history which is recorded in the CRISPR arrays.

According to CRISPR-Cas systems analysis, the hosts with the highest predicted frequencies were *Vibrio cholerae*, followed by *Enterococcus faecalis* and *Phascolarctobacterium faecium* in shrimp intestine, while the hosts with the highest predicted frequencies were *Acinetobacter baumannii* and *Geobacillus thermoleovorans* in sediment (Supplementary Table S7). The matched virus contigs mainly belonged to Caudovirales (65.70%), Microviridae (20.35%) and Myoviridae (17.44%) in shrimp intestine, and Caudovirales (75.34%), Siphoviridae (58.90%) and Myoviridae (15.07%) in sediment (Supplementary Table S7). Furthermore, most of these contigs were linked to one specific prokaryotic genus, and only 9.87% contigs were linked to two or more prokaryotic genera, which may have broad host ranges (Figure 4). According to these results of CRISPR-Cas systematic analysis, it could be inferred that symbiotic relationships existed between commensal phage and prokaryote.

In order to further investigate the effect of the relationship between phage-host interactions on microbial community, a co-occurrence network based on Spearman’s correlations was constructed to analyze relationships of the relative abundance of prokaryotes. In the shrimp intestine, there were 59 associations (edges) between 45 genera (nodes), with a much higher proportion of positive correlations (83.05%) than negative ones (16.95%). The hubs connecting mostly with members of others in the shrimp intestine systems belonged to the genus of *Escherichia*-*Shigella*, *Bacteroides*, *Acinetobacter*, *Mycobacterium,* and *Vibrio*, which were predicted as phage hosts (Supplementary Figure S6A). In the sediment, there were included 185 associations (edges) between 126 genera (nodes) in the network, with 83.78% positive correlations and 16.22% negative correlations and only one genus was predicted as phage host (Supplementary Figure S6B). These results suggested that the interaction between phages and those hosts might indirectly alter the abundance of other prokaryotes which were closely related to the phage hosts.

**Figure 5:**
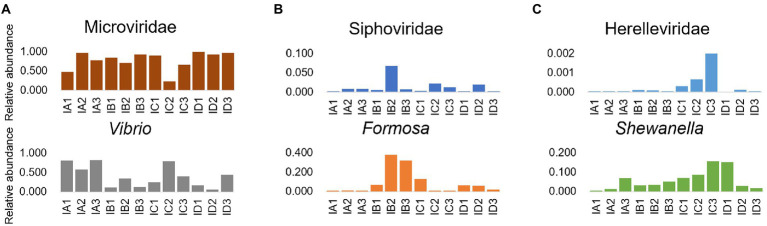
Interactional analysis of the phage and prokaryote in shrimp intestine and sediment. Relative abundance of phage (family level) and prokaryote (genus level) pairs with significant correlation (*p* < 0.05), which were dominated in the shrimp intestine microbial community, including **(A)** Microviridae and Vibrio; **(B)** Siphoviridae and Formosa; and **(C)** Herelleviridae and Shewanella.

**Figure 6:**
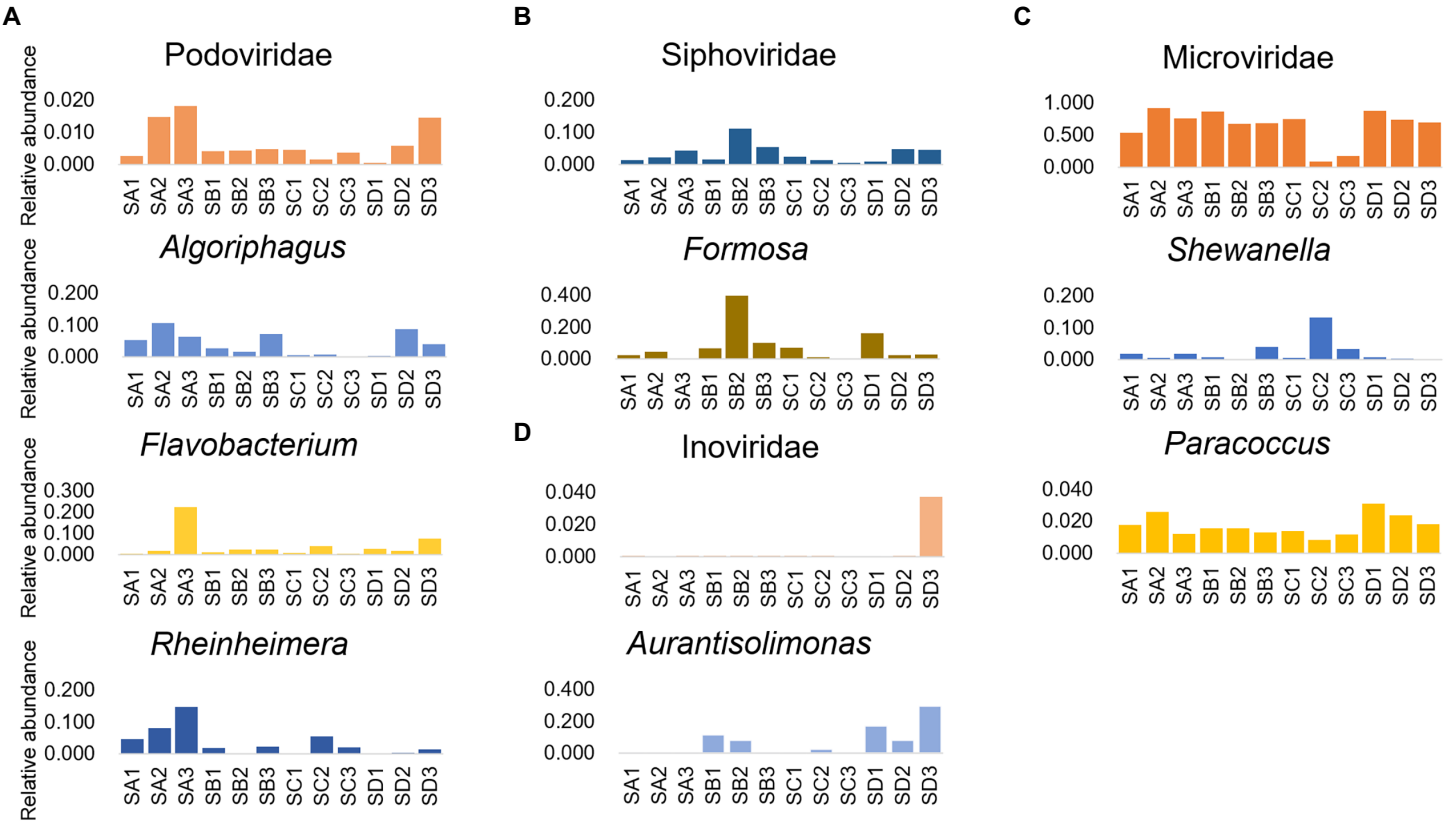
Interactional analysis of the phage and prokaryote in sediment. Relative abundance of phage (family level) and prokaryote (genus level) pairs with significant correlation (*p* < 0.05), which were dominated in the sediment microbial community, including **(A)** Podoviridae and *Algoriphagus*/*Flavobacterium*/*Rheinheimera*; **(B)** Siphoviridae and *Formosa*; **(C)** Microviridae and *Shewanella*/*Paracoccus*; and **(D)** Inoviridae and *Aurantisolimonas*.

The results of investigation on the dominant phage and prokaryote in the intestine microbial community of shrimp, revealing that the relative abundance of Microviridae and *Vibrio* showed the opposite change trend, while the relative abundance of Siphoviridae and *Formosa*, the relative abundance of Herelleviridae and *Shewanella*, showed the accordant variation trend (Figure 5). Additionally, there was a significant negative correlation between the relative abundance of Microviridae and *Vibrio* (*p* < 0.05), and significant positive correlation between the relative abundance of Siphoviridae and *Formosa* (*p* < 0.05), Herelleviridae and *Shewanella* (*p* < 0.05; Supplementary Table S8). The variation trend of the relative abundance of most of the major phages and prokaryotes in the sediment were similar, such as Podoviridae and *Algoriphagus*, *Flavobacterium*, *Rheinheimera*; Inoviridae and *Aurantisolimonas*; Microviridae and *Paracoccus* (Figure 6), and there was a significant positive correlation between their relative abundance (*p* < 0.05; Supplementary Table S8). Furthermore, the relative abundance of Microviridae and *Shewanella* showed the opposite variation trend (Figure 6C), and there was a significant negative correlation between their relative abundance (*p* < 0.05; Supplementary Table S8). These results suggested that the interaction between phages and the hosts might directly alter the abundance of prokaryotes and further mediated the microbial community in the shrimp intestine and sediment.

## Discussion

The phage-prokaryote interaction has been proved as a driver of ecological processes in different environments (Diaz-Munoz and Koskella, 2014; Koskella and Brockhurst, 2014). To identify the ecological role of phages and phage-prokaryote interaction in the shrimp intestine and sediment microhabitats, the viral and prokaryotic community profiles were investigated at four culture stages. The phage-prokaryote interactions in shrimp intestine and culture sediment can modulate the microbial community by altering the microbial community composition and function.

As a prokaryotic virus, the phage is usually studied by viral metagenomics (Huang D. et al., 2021; Dong et al., 2022). In this study, the dominant viral families in shrimp intestine were Microviridae, Circoviridae, Inoviridae, Siphoviridae, Podoviridae, and Myoviridae, which were also identified in the intestine of fish, duck, and mosquito (Fawaz et al., 2016; Filipa-Silva et al., 2020; Nebbak et al., 2021). Circoviridae is a circular single-stranded DNA (ssDNA) viral family composed of two genera, *Circovirus* and *Cyclovirus* (van Regenmortel et al., 2000), which was also frequently identified in fish, various mammals, and invertebrates (Lőrincz et al., 2011; Rosario et al., 2017). The dominant viral families in sediment were similar to that in shrimp intestine, which may be related to the fact that *L. vannamei* is typically benthic (Farfante, 1969). As with most characterized viral communities in various habitats (Liang et al., 2019; Moreno-Gallego et al., 2019; Huang D. et al., 2021), more than half of the virus in all shrimp intestine samples and in most of the sediment samples were taxonomically classified to be phage, including Microviridae, Inoviridae, Siphoviridae, Podoviridae, Myoviridae, and unclassified phages. The viral community was found to be undergone considerable changes in the early years of human life and was relatively stable in adults; moreover, the eukaryotic virus played a dominant role in the newborn gut, followed by the Caudovirales, and then the family Microviridae gradually increased and began to dominate (Lim et al., 2015). In the present study, Microviridae were the colonizers with the highest relative abundance in most of the shrimp intestine samples, rather than the microbial composition changed considerably with the culture process. The previous study proved that the viral community is closely related to the surrounding environment, especially diet (Minot et al., 2011). In addition, the prevalence of Microviridae can be somewhat exaggerated due to amplification bias (Sutton and Hill, 2019). In this study, shrimp has been fed with the same artificial feed, and the initial colonization of shrimp enterovirus may have been inherent before the start of the experiment. These results suggested that phage plays a major role in the shrimp intestine and sediment viral composition.

In the present study, Proteobacteria, Bacteroidota, Planctomycetota, and Verrucomicrobiota were the dominant phyla in shrimp intestine and sediment, which was consistent with previous studies of SCPE microbial communities (Zeng et al., 2017; Hou et al., 2018). However, the relative abundance of Cyanobacteria in shrimp intestines was low, which might be concerned with the fact that the culture ponds in this experiment were set indoors and the light intensity was lower than that in the outdoor culture environment. *Vibrio* is usually associated with a broad spectrum of diseases (Huang et al., 2016a). In our study, *Vibrio* was dominated in shrimp intestine and the relative abundance of *Vibrio* varied along four culture stages, which was consistent with the results of previous studies on the culture process of SCPE (Huang et al., 2016b; Cornejo-Granados et al., 2017). In addition, the LEfSe analysis results showed that three ASVs belonging to *Vibrio* and four viral contigs were annotated as *Vibrio* phage with significantly higher abundance in shrimp intestine at the stage C, which suggested that the phage composition seems to be closely related to the prokaryotic composition.

Prior studies suggest that the microbial community structure shifted significantly during host development in shrimp and other aquaculture animals (Li et al., 2017; Zeng et al., 2017; Wang et al., 2020). The variation of microbial community structure was closely related to multiple factors, including environment physicochemical parameters (Hou et al., 2020; Zhang et al., 2021), diet (Schulfer et al., 2020), and microbial interaction (Frey-Klett et al., 2011). Similarly, the viral and prokaryotic community structure of the shrimp intestine and sediment were significantly different at four culture stages and the Procrustes analysis revealed significant associations between the community of phage and prokaryote. Some studies indicated that interactions between phages and prokaryotes are ubiquitous in natural environment and often alter the diversity of prokaryotic populations within complex communities of microbes (Diaz-Munoz and Koskella, 2014). It may be hinted that the prokaryotic community was closely related to the phage community, and the phage-prokaryote interaction was possibly involved in mediating the microbial community in the shrimp intestine and sediment. However, the significant correlations between the α-diversity indexes of phage and prokaryote had not observed. Moreover, the pairwise distance matrices showed a weak positive correlation between phage and prokaryote β-diversity measures in sediment but not in shrimp intestine, which can be speculated that extracellular phage community diversity patterns did not mirror those of the prokaryotic community and the interaction of phage and prokaryote were multiple with different patterns.

Microbial network construction is a popular exploratory data analysis technique (Faust, 2021), which has been applied to predict biotic interactions (Coutinho et al., 2017; Chen et al., 2021). The co-occurrence networks of prokaryote-prokaryote and prokaryote-phage were constructed, respectively, in this study, with several keynotes of prokaryotes predicted corresponding phages, which suggested that prokaryote-phage interaction might indirectly alter the abundance of other prokaryotes which were closely related to the phage hosts. In some cases, each prokaryote is potentially targeted by one or more phages, resulting in a positive or negative correlation between phage-prokaryote pairs (Wigington et al., 2016). In the present study, the positive and negative link numbers of phage-prokaryote pairs in the shrimp intestine and sediment were almost same, suggesting that the interaction between phages and prokaryotes is not one way and none of them occupied an overwhelming status in the shrimp intestine and sediment microhabitat ecosystem. Thus, there may have a complex network pattern between phages and prokaryotes, which might be driven by different survival strategies of phages.

The ecological paradigm of phage life strategy is still controversial such as KtW and PtW model, while the switch between life strategies is contingent on environmental nutrients, physiological conditions of the hosts, and phage types (Zhang et al., 2017). In this study, most phages were predicted with one specific prokaryotic host, while some phages were predicted with broad host ranges. Thus, an important question to ponder is that whether different phages had different survival strategies in shrimp intestine and sediment microhabitat ecosystem. To address this concern, the relationship between phages and prokaryotes with different interaction patterns was further analyzed. The relative abundance of Microviridae and *Vibrio* showed the opposite changing trend at different culture stages. Moreover, the relative abundance of *Vibrio* and Inoviridae increased simultaneously at IC2. In addition, some Inoviridae contigs had cholera enterotoxin and closed toxin genes, which will enhance the infection ability of *Vibrio*. A previous study showed that Inoviridae has a helical structure and do not lyse the prokaryote after infection but use the host cell to continually produce progeny virions by extrusion through the host membrane (Knezevic and Adriaenssens, 2021). Therefore, it can be inferred that the fastest-growing *Vibrio* became primary targets for Microviridae phages and the Inoviridae phages assisted in the recovery of the abundance of *Vibrio* and increased their own reproductive opportunities. Furthermore, the relative abundance of Microviridae and *Shewanella* showed the opposite variation trend, while the relative abundance of Microviridae and *Paracoccus* showed the accordant variation trend in sediment at different culture stages. Some species of *Paracoccus* are known to promote growth performance, digestive enzyme activities, and disease resistance in *L. vannamei* (Adel et al., 2017), and several *Shewanella* species are opportunistic pathogens of aquatic species and humans (Cai et al., 2006; Yousfi et al., 2017). Thus, the opposite interactions between Microviridae and *Shewanella*/*Paracoccus* may play a positive role in shrimp health. We speculate that Microviridae has the potential to be applied in the prevention and control of aquaculture diseases, but it still needs to be further verified by means of culture. Furthermore, the interaction between Siphoviridae and *Formosa* in shrimp intestine was similar to that in sediment. Phages can maintain the microbial equilibrium by prey, top-down control microbial abundance (Breitbart et al., 2018), and improve the viability of prokaryotes (Li et al., 2021). There were a large number of the phage contigs annotated as genetic information processing genes and diverse AMGs in shrimp intestine and sediment microhabitat ecosystem, which suggested that phages might maintain a high abundance of host while reproducing along with the host reproduce by altering the host’s metabolism and viability. Therefore, the phage life strategy in shrimp intestine and sediment microhabitat might be unique from KtW and PtW model, more importantly, the coexistence strategy between phages and prokaryotes may mediate the microbial community of shrimp intestine and sediment.

## Conclusion

Collectively, phage played a major role in the viral composition and the phage communities were closely related to the prokaryotic communities. Moreover, the phage-prokaryote interactions can directly or indirectly modulate the microbial community composition and function. The coexistence strategies between phages and prokaryotes mediated the microbial community diversity in the intestine and sediment microhabitats of the SCPE. These findings expanded our cognization of the phage-prokaryote coexistence strategy in aquatic ecosystems from the microecological perspective and provided theoretical support for microecological prevention and control of shrimp culture health management.

## Data availability statement

The data presented in the study are deposited in the National Center of Biotechnology Information repository, https://www.ncbi.nlm.nih.gov/, accession number PRJNA796471, PRJNA797730. The names of the repository/repositories and accession number(s) can be found in the article/Supplementary material.

## Author contributions

ZD, SZ, RZ, DH, SB, LZ, QH, XL, and SW contributed to the study conception and design, material preparation, data collection, and analysis. ZD, JH, and ZH written the first draft of the manuscript. All authors read and approved the final manuscript.

## Funding

This work was financially supported by Key Research and Development Projects in Guangdong Province (2020B0202010009 and 2021B0202040001), the Southern Marine Science and Engineering Guangdong Laboratory (Zhuhai) (SML2021SP203), the earmarked fund for China Agriculture Research System (CARS-48), the China-ASEAN Maritime Cooperation Fund, China-ASEAN Center for Joint Research and Promotion of Marine Aquaculture Technology, Guangdong MEPP Fund [No. GDOE (2019) A21], and the Project of Guangdong Laboratory for Lingnan Modern Agricultural Science and Technology (2021TDQD004).

## Conflict of interest

The authors declare that the research was conducted in the absence of any commercial or financial relationships that could be construed as a potential conflict of interest.

## Publisher’s note

All claims expressed in this article are solely those of the authors and do not necessarily represent those of their affiliated organizations, or those of the publisher, the editors and the reviewers. Any product that may be evaluated in this article, or claim that may be made by its manufacturer, is not guaranteed or endorsed by the publisher.

## Footnotes

^1^ https://viralzone.expasy.org/

^2^ http://www.room220.com/crt/
