# Supplementary material for: Phage-prokaryote coexistence strategy mediates microbial community diversity in the intestine and sediment microhabitats of shrimp culture pond ecosystem
Source: Front Microbiol. 2022 Sep 23;13:1011342. doi: 10.3389/fmicb.2022.1011342 (PMC9537357; doi:10.3389/fmicb.2022.1011342)
Supplement: Supplementary file 1 [file Data_Sheet_1.docx]

Supplementary Material


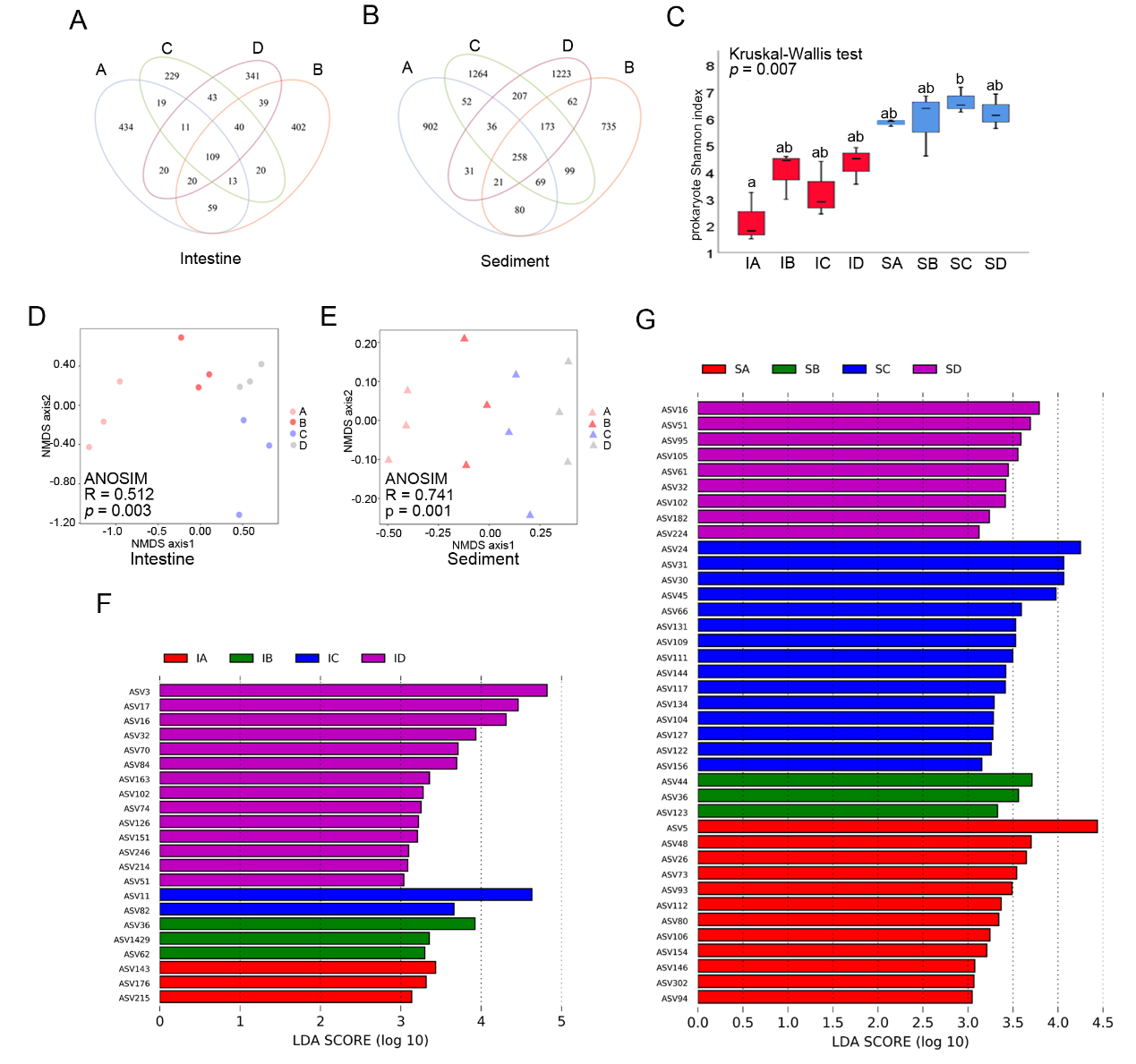


**Figure S1. Comparative analysis of shrimp intestine and sediment prokaryotic community at four culture stages.** Venn analysis of **(A)** shrimp intestine and **(B)** sediment prokaryotic communities (ASV level) at four culture stages (stage A, stage B, stage C and stage D). **(C)** Box plots figure showed the range of prokaryotic Shannon index in shrimp intestine (red) and sediment (blue) at four culture stages. The significant difference calculated by the Kruskal-Wallis test was shown by marking a, ab and b. The β-diversity of prokaryotic communities of **(D)** shrimp intestine and **(E)** sediment at four culture stages was analyzed by NMDS and ANOSIM based on the Bray-Curtis distance. LEfSe analysis showed differentially abundant prokaryotes (ASV level) among four culture stages in **(F)** shrimp intestine and **(G)** sediment based on *P* < 0.05 and LDA score > 3.0.


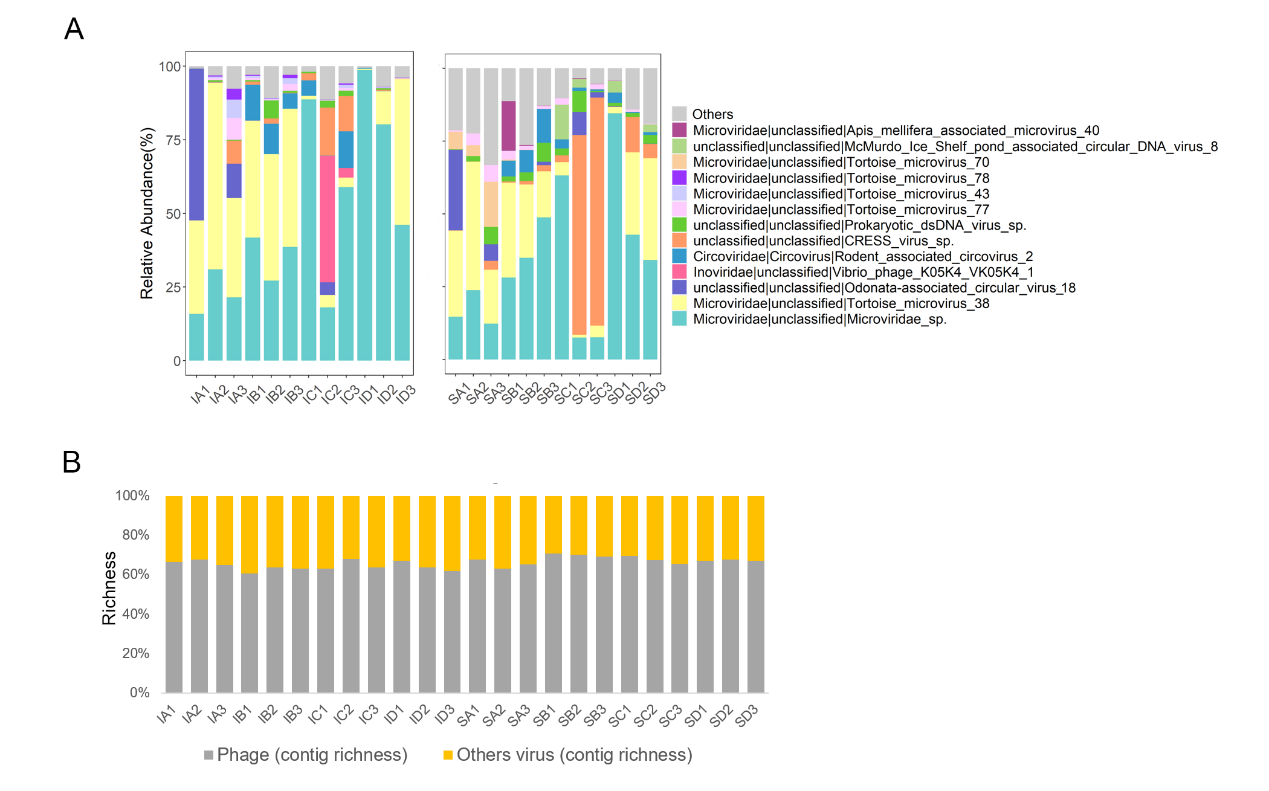


**Figure S2. Composition analysis of virus community in shrimp intestine and sediment. (A)** Relative abundances of dominant viral species. **(B)** The percentage of the phage and other virus richness in shrimp intestine and sediment viral community.


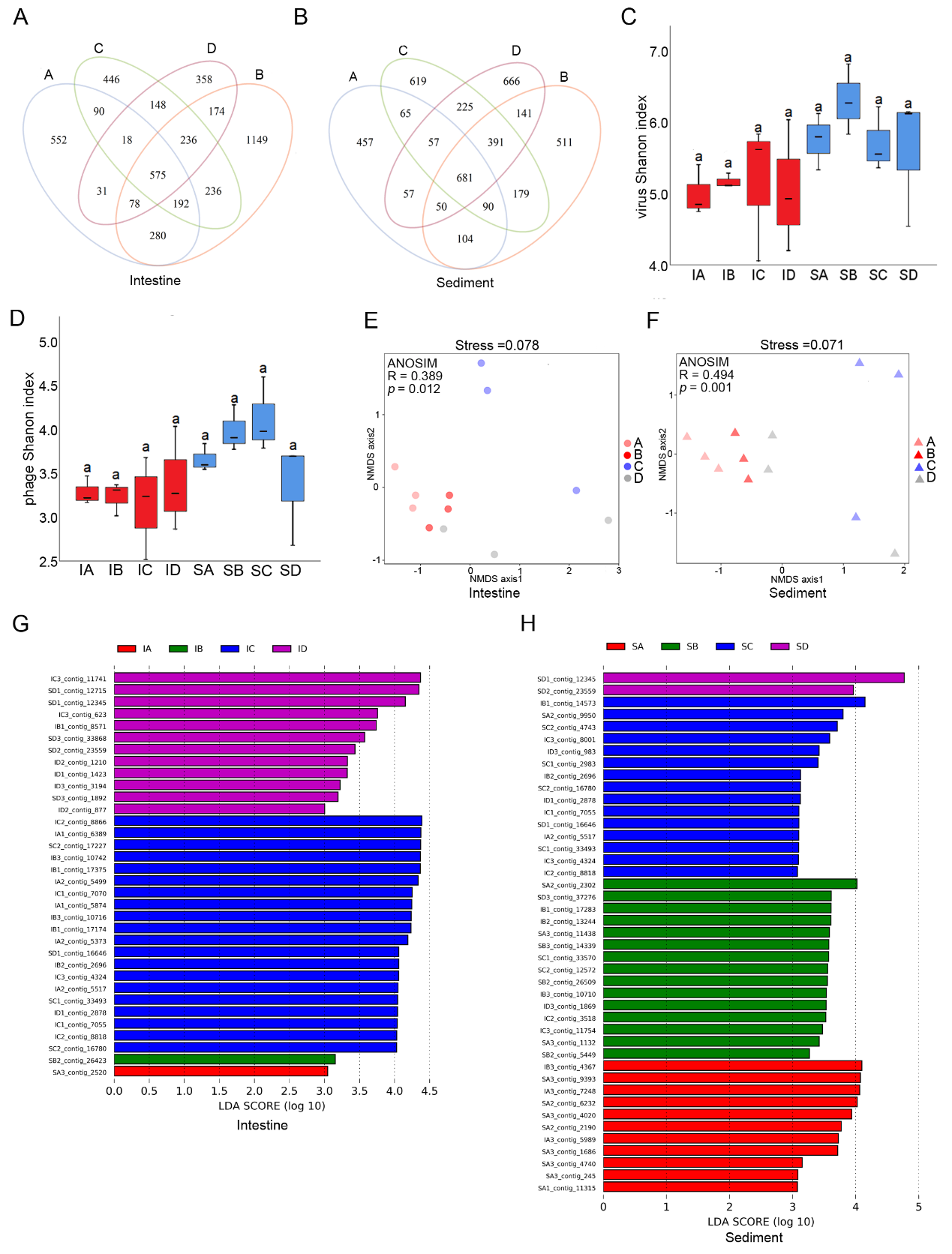


**Figure S3. Comparative analysis of shrimp intestine and sediment viral community at four culture stages.** Venn analysis of **(A)** shrimp intestine and **(B)** sediment viral communities (contig level) at four culture stages (stage A, stage B, stage C and stage D). Box plots figure showed the range of **(C)** virus Shannon index and **(D)** phage Shannon index in shrimp intestine (red) and sediment (blue) at four culture stages. The significant difference calculated by the Kruskal-Wallis test was shown by marking a. The β-diversity of viral communities of **(E)** shrimp intestine and **(F)** sediment at four culture stages was analyzed by NMDS and ANOSIM based on the Bray-Curtis distance. LEfSe analysis showed differentially abundant viruses (contig level) among four culture stages in **(G)** shrimp intestine and **(H)** sediment based on *P* < 0.05 and LDA score > 3.0.


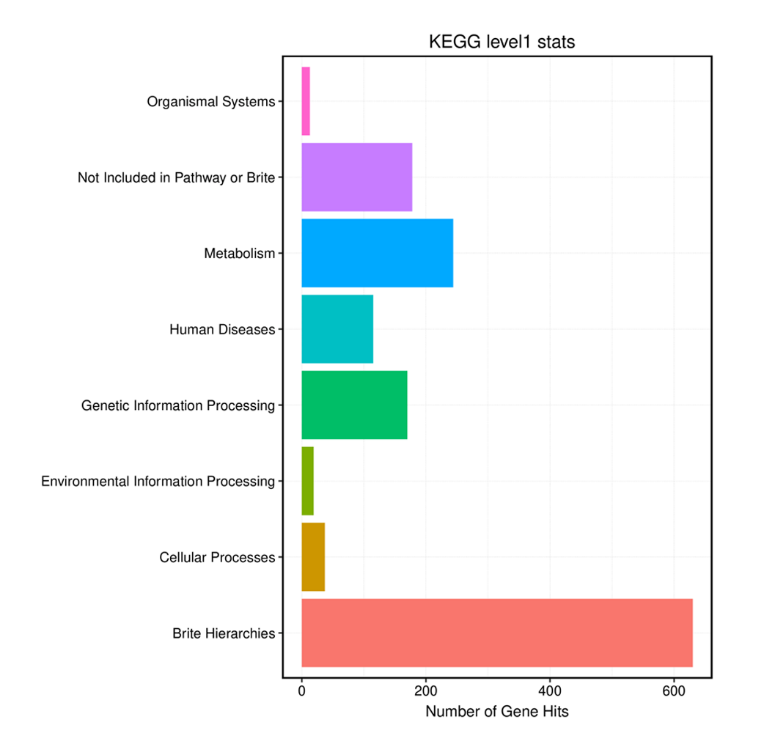


**Figure S4. Functional analysis of viral community.** The number of unigene hit with KEGG level 1 in the viral community of shrimp intestine and the sediment.


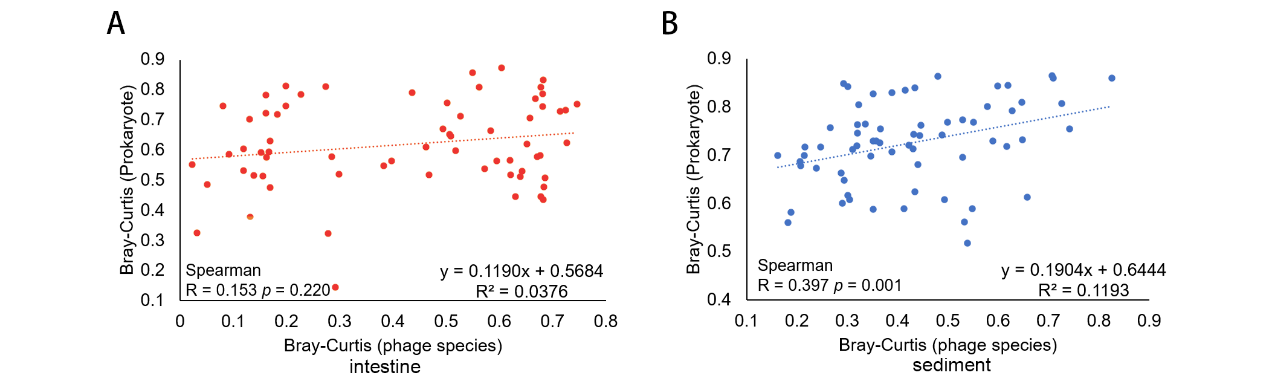


**Figure S5. Correlations analysis of the microbial community in shrimp intestine and sediment.** Plot showed the relationship between the Bray-Curtis distance of phage (species level) and prokaryotic (ASV level) community in **(A)** shrimp intestine and **(B)** sediment, and the Spearman correlation coefficient was indicated.


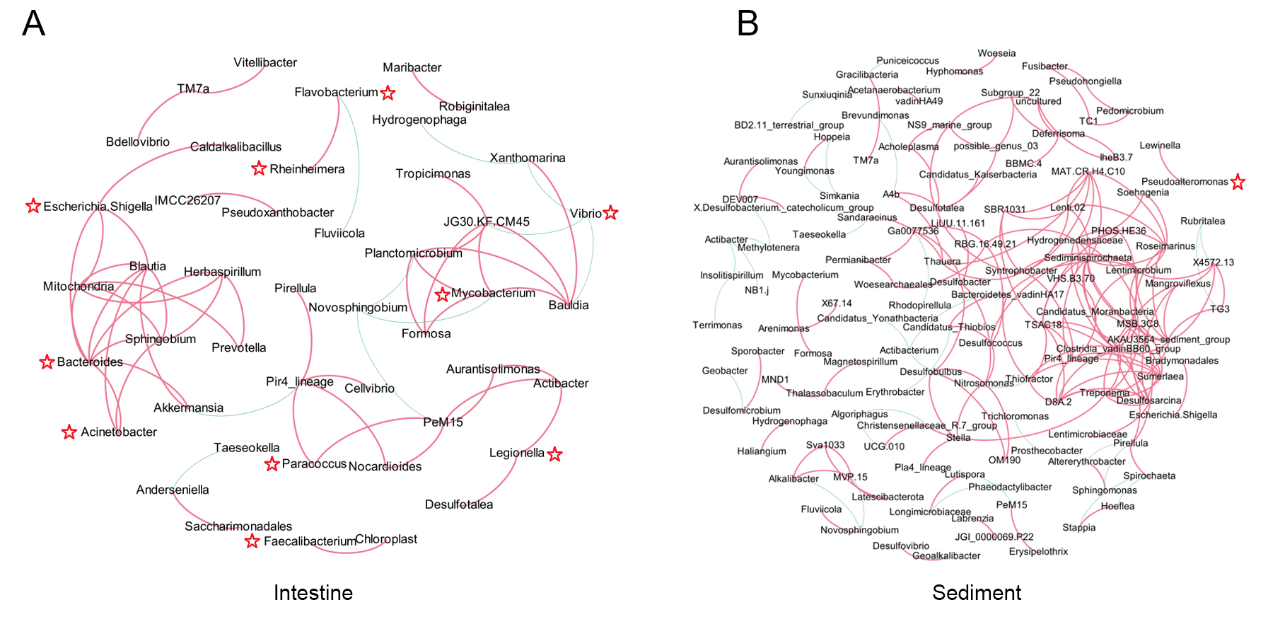


**Figure S6. Analysis of the role of the relationship between phage-host interactions on the microbial community.** Co-occurrence network of **(A)** shrimp intestine prokaryotic community (genus level) and **(B)** sediment prokaryotic community (genus level). The positive or negative linkages of the association networks were based on positive or negative Spearman’s correlations between any pairs of nodes. The red line means positive correlations, and the green line means negative correlations, and the star indicated that prokaryote was predicted as phage host.

**Table S1. Group information of sample collection.**

| Stage | pond1 | pond2 | pond3 | pond4 | pond5 | pond6 | pond7 | pond8 | pond9 | pond10 | pond11 | pond12 |
| --- | --- | --- | --- | --- | --- | --- | --- | --- | --- | --- | --- | --- |
| stage A | Intestine sample A1  (IA1), Sediment sample A1  (SA1) | Intestine sample A2  (IA2), Sediment sample A2  (SA2) | Intestine sample A3  (IA3), Sediment sample A3  (SA3) |  |  |  |  |  |  |  |  |  |
| stage B |  |  |  | Intestine sample B1  (IB1), Sediment sample B1  (SB1) | Intestine sample B2  (IB2), Sediment sample B2  (SB2) | Intestine sample B3  (IB3), Sediment sample B3  (SB3) |  |  |  |  |  |  |
| stage C |  |  |  |  |  |  | Intestine sample C1  (IC1), Sediment sample C1  (SC1) | Intestine sample C2  (IC2), Sediment sample C2  (SC2) | Intestine sample C3  (IC3), Sediment sample C3  (SC3) |  |  |  |
| stage D |  |  |  |  |  |  |  |  |  | Intestine sample D1  (ID1), Sediment sample D1  (SD1) | Intestine sample D2  (ID2), Sediment sample D2  (SD2) | Intestine sample D3  (ID3), Sediment sample D3  (SD3) |

There were 12 shrimp intestine samples and 12 cultural sediment samples collected from 12 ponds. Every 15 days in 4 culture stages (from stage A to D), three repeat intestinal samples (from 1 to 3) and three repeat sediment samples (from 1 to 3) were collected from three shrimp culture ponds.

| **Table S2.** α-**diversity analysis of shrimp intestine and sediment.** | | | | | | | | | | |  |
| --- | --- | --- | --- | --- | --- | --- | --- | --- | --- | --- | --- |
| group | viral richness (contig) | viral Shannon index (contig) | phage richness (contig） | phage Shannon index (contig) | viral richness (species) | viral Shannon index (species) | phage richness (species) | phage Shannon index (species) | procaryotic richness  (ASV) | procaryotic Shannon index  (ASV) | |
| IA1 | 838 | 3.36 | 558 | 3.16 | 275 | 1.07 | 196 | 0.75 | 316 | 1.80 | |
| IA2 | 969 | 3.29 | 657 | 3.21 | 289 | 1.00 | 192 | 0.90 | 375 | 3.24 | |
| IA3 | 1351 | 3.74 | 878 | 3.47 | 440 | 2.08 | 325 | 1.71 | 252 | 1.51 | |
| IB1 | 2056 | 3.54 | 1250 | 3.31 | 587 | 1.37 | 352 | 0.97 | 470 | 4.58 | |
| IB2 | 1476 | 3.66 | 942 | 3.01 | 486 | 1.78 | 334 | 1.2 | 234 | 2.98 | |
| IB3 | 1359 | 3.54 | 860 | 3.37 | 386 | 1.33 | 249 | 1.11 | 269 | 4.43 | |
| IC1 | 952 | 2.81 | 603 | 2.51 | 288 | 0.58 | 184 | 0.19 | 227 | 4.40 | |
| IC2 | 1128 | 3.89 | 770 | 3.23 | 327 | 1.99 | 224 | 1.38 | 228 | 2.44 | |
| IC3 | 1251 | 4.04 | 801 | 3.67 | 405 | 1.61 | 274 | 0.96 | 220 | 2.89 | |
| ID1 | 632 | 2.91 | 425 | 2.86 | 152 | 0.10 | 104 | 0.04 | 331 | 4.50 | |
| ID2 | 941 | 4.18 | 602 | 4.03 | 257 | 0.90 | 153 | 0.68 | 345 | 4.91 | |
| ID3 | 1221 | 3.41 | 758 | 3.27 | 335 | 0.92 | 211 | 0.77 | 251 | 3.55 | |
| SA1 | 811 | 4.01 | 551 | 3.54 | 317 | 1.80 | 259 | 1.42 | 655 | 5.88 | |
| SA2 | 848 | 3.69 | 536 | 3.59 | 284 | 1.89 | 222 | 1.78 | 581 | 5.94 | |
| SA3 | 948 | 4.24 | 621 | 3.83 | 335 | 2.71 | 277 | 2.45 | 759 | 5.71 | |
| SB1 | 1238 | 4.04 | 879 | 3.77 | 467 | 2.02 | 372 | 1.71 | 707 | 6.38 | |
| SB2 | 1447 | 4.34 | 1017 | 3.90 | 519 | 2.28 | 419 | 1.86 | 540 | 4.60 | |
| SB3 | 1027 | 4.72 | 713 | 4.27 | 372 | 2.00 | 294 | 1.38 | 795 | 6.83 | |
| SC1 | 1590 | 4.3 | 1107 | 3.78 | 565 | 1.67 | 455 | 1.07 | 956 | 6.50 | |
| SC2 | 1032 | 3.84 | 696 | 4.59 | 369 | 1.32 | 287 | 1.61 | 940 | 6.24 | |
| SC3 | 1075 | 3.71 | 708 | 3.97 | 400 | 1.03 | 302 | 1.94 | 1038 | 7.17 | |
| SD1 | 1037 | 3.14 | 698 | 2.68 | 373 | 0.79 | 285 | 0.28 | 865 | 6.11 | |
| SD2 | 1249 | 4.23 | 847 | 3.69 | 454 | 1.83 | 354 | 1.29 | 1122 | 6.91 | |
| SD3 | 1537 | 4.25 | 1032 | 3.69 | 555 | 2.06 | 426 | 1.45 | 738 | 5.63 | |

The viral (contig and species), phage (contig and species), prokaryotic (ASV) community richness, and Shannon index for α-diversity analysis of 12 shrimp intestine samples and 12 sediment samples.

**Table S3.** **Linear discriminant analysis of enriched prokaryotic taxonomies at four culture stages in shrimp intestine and sediment using LEfSe.**

| sample | Group | ASV | LDA SCORE  (log 10) | Sig. | Taxonomy |
| --- | --- | --- | --- | --- | --- |
| Intestine | IA | ASV 143 | 3.43 | 0.042 | d_Bacteria; p_Firmicutes; c_Bacilli; o_Lactobacillales; f_Enterococcaceae; g_Enterococcus |
|  | IA | ASV 176 | 3.31 | 0.042 | d_Bacteria; p_Bacteroidota; c_Bacteroidia; o_Bacteroidales; f_Tannerellaceae |
|  | IA | ASV 215 | 3.14 | 0.013 | d_Bacteria; p_Proteobacteria; c_Alphaproteobacteria; o_Rhodobacterales; f_Rhodobacteraceae |
|  | IB | ASV 36 | 3.92 | 0.026 | d_Bacteria; p_Planctomycetota; c_Planctomycetes; o_Planctomycetales |
|  | IB | ASV 1429 | 3.35 | 0.013 | d_Bacteria; p_Actinobacteriota; c_Actinobacteria; o_PeM15; f_PeM15; g_PeM15 |
|  | IB | ASV 62 | 3.30 | 0.033 | d_Bacteria; p_Actinobacteriota; c_Actinobacteria; o_Corynebacteriales; f_Mycobacteriaceae; g_Mycobacterium |
|  | IC | ASV 11 | 4.63 | 0.040 | d_Bacteria; p_Proteobacteria; c_Gammaproteobacteria; o_Vibrionales; f_Vibrionaceae; g_Vibrio |
|  | IC | ASV 82 | 3.66 | 0.025 | d_Bacteria; p_Proteobacteria; c_Gammaproteobacteria; o_Vibrionales; f_Vibrionaceae; g_Vibrio; s_Vibrio_cholerae |
|  | ID | ASV 3 | 4.82 | 0.044 | d_Bacteria; p_Bacteroidota; c_Bacteroidia; o_Chitinophagales; f_Chitinophagaceae; g_Aurantisolimonas; s_uncultured_bacterium |
|  | ID | ASV 17 | 4.46 | 0.043 | d_Bacteria; p_Proteobacteria; c_Alphaproteobacteria; o_Rhodobacterales; f_Rhodobacteraceae; g_Paracoccus |
|  | ID | ASV 16 | 4.31 | 0.037 | d_Bacteria; p_Planctomycetota; c_Planctomycetes; o_Pirellulales; f_Pirellulaceae; g_Pirellula; s_uncultured_organism |
|  | ID | ASV 32 | 3.93 | 0.041 | d_Bacteria; p_Planctomycetota; c_Planctomycetes; o_Pirellulales; f_Pirellulaceae; g_Pirellula |
|  | ID | ASV 70 | 3.71 | 0.025 | d_Bacteria; p_Proteobacteria; c_Alphaproteobacteria; o_Rhodobacterales; f_Rhodobacteraceae; g_Paracoccus |
|  | ID | ASV 84 | 3.69 | 0.025 | d_Bacteria; p_Actinobacteriota; c_Actinobacteria; o_PeM15; f_PeM15; g_PeM15; s_metagenome |
|  | ID | ASV 163 | 3.36 | 0.013 | d_Bacteria; p_Verrucomicrobiota; c_Verrucomicrobiae; o_Verrucomicrobiales; f_Rubritaleaceae; g_Rubritalea |
|  | ID | ASV 102 | 3.28 | 0.025 | d_Bacteria; p_Bacteroidota; c_Bacteroidia; o_Flavobacteriales; f_Flavobacteriaceae; g_Actibacter |
|  | ID | ASV 74 | 3.25 | 0.031 | d_Bacteria; p_Actinobacteriota; c_Actinobacteria; o_PeM15; f_PeM15; g_PeM15 |
|  | ID | ASV 126 | 3.22 | 0.031 | d_Bacteria; p_Verrucomicrobiota; c_Verrucomicrobiae; o_Verrucomicrobiales; f_Rubritaleaceae; g_Haloferula; s_uncultured_Verrucomicrobia |
|  | ID | ASV 151 | 3.21 | 0.024 | d_Bacteria; p_Planctomycetota; c_Planctomycetes; o_Pirellulales; f_Pirellulaceae; g_Rhodopirellula; s_Pirellula_sp. |
|  | ID | ASV 246 | 3.10 | 0.013 | d_Bacteria; p_Verrucomicrobiota; c_Chlamydiae; o_Chlamydiales; f_Simkaniaceae; g_uncultured |
|  | ID | ASV 214 | 3.09 | 0.042 | d_Bacteria; p_Planctomycetota; c_Planctomycetes; o_Pirellulales; f_Pirellulaceae; g_Pir4_lineage |
|  | ID | ASV 51 | 3.04 | 0.032 | d_Bacteria; p_Proteobacteria; c_Gammaproteobacteria; o_Cellvibrionales; f_Cellvibrionaceae; g_Cellvibrio |
| sediment | SA | ASV 5 | 4.437 | 0.022 | d_Bacteria; p_Bacteroidota; c_Bacteroidia; o_Cytophagales; f_Cyclobacteriaceae; g_Algoriphagus |
|  | SA | ASV 48 | 3.701 | 0.016 | d_Bacteria; p_Actinobacteriota; c_Actinobacteria; o_Micrococcales; f_Demequinaceae |
|  | SA | ASV 26 | 3.647 | 0.033 | d_Bacteria; p_Proteobacteria; c_Alphaproteobacteria; o_Rhodobacterales; f_Rhodobacteraceae; g_Paracoccus |
|  | SA | ASV 73 | 3.540 | 0.024 | d_Bacteria; p_Proteobacteria; c_Alphaproteobacteria; o_Sphingomonadales; f_Sphingomonadaceae; g_Erythrobacter |
|  | SA | ASV 93 | 3.490 | 0.024 | d_Bacteria; p_Bacteroidota; c_Bacteroidia; o_Flavobacteriales; f_Flavobacteriaceae |
|  | SA | ASV 112 | 3.370 | 0.031 | d_Bacteria; p_Proteobacteria; c_Alphaproteobacteria; o_Rhodobacterales; f_Rhodobacteraceae |
|  | SA | ASV 80 | 3.342 | 0.043 | d_Bacteria; p_Proteobacteria; c_Alphaproteobacteria; o_Rhodobacterales; f_Rhodobacteraceae; g_Gemmobacter |
|  | SA | ASV 106 | 3.243 | 0.041 | d_Bacteria; p_Proteobacteria; c_Alphaproteobacteria; o_Rhodobacterales; f_Rhodobacteraceae; g_Paracoccus |
|  | SA | ASV 154 | 3.208 | 0.039 | d_Bacteria; p_Proteobacteria; c_Alphaproteobacteria; o_Rhodobacterales; f_Rhodobacteraceae |
|  | SA | ASV 146 | 3.075 | 0.036 | d_Bacteria; p_Proteobacteria; c_Alphaproteobacteria; o_Rhodobacterales; f_Rhodobacteraceae |
|  | SA | ASV 302 | 3.065 | 0.025 | d_Bacteria; p_Bacteroidota; c_Bacteroidia; o_Cytophagales; f_Spirosomaceae; g_uncultured; s_uncultured_bacterium |
|  | SA | ASV 94 | 3.046 | 0.037 | d_Bacteria; p_Proteobacteria; c_Gammaproteobacteria; o_Gammaproteobacteria_Incertae_Sedis; f_Unknown_Family; g_Unknown_Family; s_uncultured_soil |
|  | SB | ASV 44 | 3.711 | 0.028 | d_Bacteria; p_Proteobacteria; c_Alphaproteobacteria; o_Rhodobacterales; f_Rhodobacteraceae |
|  | SB | ASV 36 | 3.561 | 0.028 | d_Bacteria; p_Planctomycetota; c_Planctomycetes; o_Planctomycetales |
|  | SB | ASV 123 | 3.327 | 0.041 | d_Bacteria; p_Proteobacteria; c_Alphaproteobacteria |
|  | SC | ASV 24 | 4.248 | 0.050 | d_Bacteria; p_Bacteroidota; c_Bacteroidia; o_Chitinophagales; f_Saprospiraceae; g_uncultured |
|  | SC | ASV 31 | 4.064 | 0.027 | d_Bacteria; p_Bacteroidota; c_Bacteroidia; o_Bacteroidales; f_Prolixibacteraceae; g_uncultured |
|  | SC | ASV 30 | 4.064 | 0.038 | d_Bacteria; p_Spirochaetota; c_Spirochaetia; o_Spirochaetales; f_Spirochaetaceae; g_Sediminispirochaeta; s_uncultured_bacterium |
|  | SC | ASV 45 | 3.978 | 0.025 | d_Bacteria; p_Bacteroidota; c_Bacteroidia; o_Sphingobacteriales; f_Lentimicrobiaceae; g_Lentimicrobium |
|  | SC | ASV 66 | 3.593 | 0.024 | d_Bacteria; p_Patescibacteria; c_Parcubacteria; o_Candidatus_Moranbacteria; f_Candidatus_Moranbacteria; g_Candidatus_Moranbacteria; s_uncultured_bacterium |
|  | SC | ASV 131 | 3.529 | 0.022 | d_Bacteria; p_Bacteroidota; c_Bacteroidia; o_Bacteroidales; f_Prolixibacteraceae; g_Roseimarinus; s_metagenome |
|  | SC | ASV 109 | 3.529 | 0.025 | d_Bacteria; p_Desulfobacterota; c_Desulfobacteria; o_Desulfobacterales; f_Desulfococcaceae; g_Desulfococcus; s_uncultured_bacterium |
|  | SC | ASV 111 | 3.497 | 0.024 | d_Bacteria; p_Proteobacteria; c_Gammaproteobacteria; o_Burkholderiales; f_Rhodocyclaceae; g_Thauera |
|  | SC | ASV 144 | 3.419 | 0.014 | d_Bacteria; p_Spirochaetota; c_Spirochaetia; o_Spirochaetales; f_Spirochaetaceae; g_Sediminispirochaeta; s_uncultured_bacterium |
|  | SC | ASV 117 | 3.414 | 0.015 | d_Bacteria; p_Bacteroidota; c_Bacteroidia; o_Bacteroidales; f_Prolixibacteraceae; g_Draconibacterium; s_uncultured_bacterium |
|  | SC | ASV 134 | 3.291 | 0.022 | d_Bacteria; p_Chloroflexi; c_Anaerolineae; o_Anaerolineales; f_Anaerolineaceae; g_uncultured; s_uncultured_bacterium |
|  | SC | ASV 104 | 3.281 | 0.041 | d_Bacteria; p_Proteobacteria; c_Alphaproteobacteria; o_Rhodobacterales; f_Rhodobacteraceae |
|  | SC | ASV 127 | 3.275 | 0.031 | d_Bacteria; p_Proteobacteria; c_Alphaproteobacteria; o_Rhodobacterales; f_Rhodobacteraceae; g_Paracoccus |
|  | SC | ASV 122 | 3.258 | 0.023 | d_Bacteria; p_Bacteroidota; c_Bacteroidia; o_Bacteroidales; f_Bacteroidetes_vadinHA17; g_Bacteroidetes_vadinHA17; s_uncultured_bacterium |
|  | SC | ASV 156 | 3.153 | 0.028 | d_Bacteria; p_Desulfobacterota; c_Desulfobulbia; o_Desulfobulbales; f_Desulfocapsaceae |
|  | SD | ASV 16 | 3.792 | 0.015 | d_Bacteria; p_Planctomycetota; c_Planctomycetes; o_Pirellulales; f_Pirellulaceae; g_Pirellula; s_uncultured_organism |
|  | SD | ASV 51 | 3.692 | 0.027 | d_Bacteria; p_Proteobacteria; c_Gammaproteobacteria; o_Cellvibrionales; f_Cellvibrionaceae; g_Cellvibrio |
|  | SD | ASV 95 | 3.588 | 0.022 | d_Bacteria; p_Spirochaetota; c_Spirochaetia; o_Spirochaetales; f_Spirochaetaceae; g_Sediminispirochaeta; s_uncultured_bacterium |
|  | SD | ASV 105 | 3.554 | 0.030 | d_Bacteria; p_Proteobacteria; c_Gammaproteobacteria; o_Burkholderiales; f_Rhodocyclaceae |
|  | SD | ASV 61 | 3.447 | 0.041 | d_Bacteria; p_Bacteroidota; c_Bacteroidia; o_Flavobacteriales; f_Flavobacteriaceae; g_Actibacter; s_uncultured_bacterium |
|  | SD | ASV 32 | 3.417 | 0.031 | d_Bacteria; p_Planctomycetota; c_Planctomycetes; o_Pirellulales; f_Pirellulaceae; g_Pirellula |
|  | SD | ASV 102 | 3.415 | 0.025 | d_Bacteria; p_Bacteroidota; c_Bacteroidia; o_Flavobacteriales; f_Flavobacteriaceae; g_Actibacter |
|  | SD | ASV 182 | 3.237 | 0.025 | d_Bacteria; p_Proteobacteria; c_Gammaproteobacteria |
|  | SD | ASV 224 | 3.123 | 0.037 | d_Bacteria; p_Verrucomicrobiota; c_Verrucomicrobiae; o_Opitutales; f_Opitutaceae; g_Lacunisphaera; s_metagenome |

LEfSe analysis showed differentially abundant prokaryotes (ASV level) among four culture stages in shrimp intestine and sediment based on *P* < 0.05 and LDA score > 3.0.

**Table S4** **Linear discriminant analysis of enriched viral taxonomies at four culture stages in shrimp intestine and sediment using LEfSe.**

| sample | group | Contig | LDA SCORE  (log 10) | Sig. | Taxonomy | Host |
| --- | --- | --- | --- | --- | --- | --- |
| intestine | IA | SA3_contig_2520 | 3.046 | 0.025 | Siphoviridae\|unclassified\|Arthrobacter phage Seahorse | / |
|  | IB | SB2_contig_26423 | 3.153 | 0.046 | Siphoviridae\|unclassified\|Flavobacterium phage vB_FspS_stinky9-1 | / |
|  | IC | IC2_contig_8866 | 4.390 | 0.034 | Microviridae\|unclassified\|Microviridae sp. | / |
|  | IC | IA1_contig_6389 | 4.377 | 0.034 | Microviridae\|unclassified\|Microviridae sp. | / |
|  | IC | SC2_contig_17227 | 4.373 | 0.033 | Microviridae\|unclassified\|Microviridae sp. | / |
|  | IC | IB3_contig_10742 | 4.368 | 0.034 | Microviridae\|unclassified\|Microviridae sp. | / |
|  | IC | IB1_contig_17375 | 4.367 | 0.034 | Microviridae\|unclassified\|Microviridae sp. | / |
|  | IC | IA2_contig_5499 | 4.337 | 0.034 | Microviridae\|unclassified\|Microviridae sp. | / |
|  | IC | IC1_contig_7070 | 4.250 | 0.034 | Inoviridae\|unclassified\|Vibrio phage K05K4_VK05K4_1 | Vibrio/metschnikovii |
|  | IC | IA1_contig_5874 | 4.245 | 0.034 | Inoviridae\|unclassified\|Vibrio phage K05K4_VK05K4_1 | / |
|  | IC | IB3_contig_10716 | 4.237 | 0.034 | Inoviridae\|unclassified\|Vibrio phage K05K4_VK05K4_1 | / |
|  | IC | IB1_contig_17174 | 4.234 | 0.036 | Inoviridae\|unclassified\|Vibrio phage K05K4_VK05K4_1 | / |
|  | IC | IA2_contig_5373 | 4.189 | 0.034 | Inoviridae\|unclassified\|Vibrio phage K05K4_VK05K4_1 | / |
|  | IC | SD1_contig_16646 | 4.060 | 0.026 | Microviridae\|unclassified\|Microviridae sp. | / |
|  | IC | IB2_contig_2696 | 4.059 | 0.026 | Microviridae\|unclassified\|Microviridae sp. | / |
|  | IC | IC3_contig_4324 | 4.057 | 0.026 | Microviridae\|unclassified\|Microviridae sp. | / |
|  | IC | IA2_contig_5517 | 4.051 | 0.026 | Microviridae\|unclassified\|Microviridae sp. | / |
|  | IC | SC1_contig_33493 | 4.044 | 0.026 | Microviridae\|unclassified\|Microviridae sp. | / |
|  | IC | ID1_contig_2878 | 4.043 | 0.026 | Microviridae\|unclassified\|Microviridae sp. | / |
|  | IC | IC1_contig_7055 | 4.038 | 0.026 | Microviridae\|unclassified\|Microviridae sp. | / |
|  | IC | IC2_contig_8818 | 4.031 | 0.026 | Microviridae\|unclassified\|Microviridae sp. | / |
|  | IC | SC2_contig_16780 | 4.029 | 0.026 | Microviridae\|unclassified\|Microviridae sp. | / |
|  | ID | IC3_contig_11741 | 4.371 | 0.034 | Microviridae\|unclassified\|Microviridae sp. | / |
|  | ID | SD1_contig_12715 | 4.348 | 0.036 | Microviridae\|unclassified\|Microviridae sp. | / |
|  | ID | SD1_contig_12345 | 4.153 | 0.033 | Microviridae\|unclassified\|Microviridae sp. | / |
|  | ID | IC3_contig_623 | 3.756 | 0.043 | Microviridae\|unclassified\|Microviridae sp. | / |
|  | ID | IB1_contig_8571 | 3.740 | 0.041 | Microviridae\|unclassified\|Microviridae sp. | / |
|  | ID | SD3_contig_33868 | 3.574 | 0.030 | Microviridae\|unclassified\|Microviridae sp. | / |
|  | ID | SD2_contig_23559 | 3.435 | 0.027 | Microviridae\|unclassified\|Microviridae sp. | / |
|  | ID | ID2_contig_1210 | 3.327 | 0.022 | Microviridae\|unclassified\|Microviridae sp. | / |
|  | ID | ID1_contig_1423 | 3.325 | 0.038 | Microviridae\|unclassified\|Microviridae sp. | / |
|  | ID | ID3_contig_3194 | 3.222 | 0.037 | Microviridae\|unclassified\|Microviridae sp. | / |
|  | ID | SD3_contig_1892 | 3.192 | 0.024 | Microviridae\|unclassified\|Microviridae sp. | / |
|  | ID | ID2_contig_877 | 3.004 | 0.025 | Microviridae\|unclassified\|Microviridae sp. | / |
| sediment | SA | IB3_contig_4367 | 4.099 | 0.042 | Microviridae\|unclassified\|Tortoise microvirus 46 | / |
|  | SA | SA3_contig_9393 | 4.075 | 0.042 | Microviridae\|unclassified\|Tortoise microvirus 37 | / |
|  | SA | IA3_contig_7248 | 4.064 | 0.042 | Microviridae\|unclassified\|Tortoise microvirus 70 | / |
|  | SA | SA2_contig_6232 | 4.025 | 0.042 | Microviridae\|unclassified\|Tortoise microvirus 37 | / |
|  | SA | SA3_contig_4020 | 3.937 | 0.042 | Microviridae\|unclassified\|Tortoise microvirus 46 | / |
|  | SA | SA2_contig_2190 | 3.772 | 0.042 | Podoviridae\|Jwalphavirus\|Achromobacter phage vB_AxyP_19-32_Axy12 | / |
|  | SA | IA3_contig_5989 | 3.725 | 0.036 | Microviridae\|unclassified\|Tortoise microvirus 46 | / |
|  | SA | SA3_contig_1686 | 3.715 | 0.036 | Microviridae\|unclassified\|Tortoise microvirus 70 | / |
|  | SA | SA3_contig_4740 | 3.152 | 0.025 | Siphoviridae\|Camvirus\|Streptomyces phage Yosif | / |
|  | SA | SA3_contig_245 | 3.083 | 0.035 | Myoviridae\|unclassified\|Vibrio phage CP-T1 | / |
|  | SA | SA1_contig_11315 | 3.076 | 0.025 | Siphoviridae\|Pepyhexavirus\|Rhodococcus virus Pepy6 | / |
|  | SB | SA2_contig_2302 | 4.020 | 0.022 | Microviridae\|unclassified\|Microviridae sp. | / |
|  | SB | SD3_contig_37276 | 3.612 | 0.047 | Microviridae\|unclassified\|Apis mellifera associated microvirus 40 | / |
|  | SB | IB1_contig_17283 | 3.610 | 0.047 | Microviridae\|unclassified\|Apis mellifera associated microvirus 40 | / |
|  | SB | IB2_contig_13244 | 3.609 | 0.047 | Microviridae\|unclassified\|Apis mellifera associated microvirus 40 | / |
|  | SB | SA3_contig_11438 | 3.584 | 0.047 | Microviridae\|unclassified\|Apis mellifera associated microvirus 40 | / |
|  | SB | SB3_contig_14339 | 3.575 | 0.047 | Microviridae\|unclassified\|Apis mellifera associated microvirus 40 | / |
|  | SB | SC1_contig_33570 | 3.573 | 0.047 | Microviridae\|unclassified\|Apis mellifera associated microvirus 40 | / |
|  | SB | SC2_contig_12572 | 3.556 | 0.038 | Microviridae\|unclassified\|Apis mellifera associated microvirus 38 | / |
|  | SB | SB2_contig_26509 | 3.553 | 0.047 | Microviridae\|unclassified\|Apis mellifera associated microvirus 40 | / |
|  | SB | IB3_contig_10710 | 3.536 | 0.047 | Microviridae\|unclassified\|Apis mellifera associated microvirus 40 | / |
|  | SB | ID3_contig_1869 | 3.533 | 0.022 | Microviridae\|unclassified\|Microviridae sp. | / |
|  | SB | IC2_contig_3518 | 3.525 | 0.022 | Microviridae\|unclassified\|Microviridae sp. | / |
|  | SB | IC3_contig_11754 | 3.473 | 0.022 | Microviridae\|unclassified\|Microviridae sp. | / |
|  | SB | SA3_contig_1132 | 3.422 | 0.022 | Microviridae\|unclassified\|Eel River basin pequenovirus | / |
|  | SB | SB2_contig_5449 | 3.267 | 0.038 | Siphoviridae\|unclassified\|Microbacterium phage Count | / |
|  | SC | IB1_contig_14573 | 4.147 | 0.050 | Microviridae\|unclassified\|Microviridae sp. | / |
|  | SC | SA2_contig_9950 | 3.799 | 0.041 | Microviridae\|unclassified\|Microviridae sp. | / |
|  | SC | SC2_contig_4743 | 3.708 | 0.043 | Microviridae\|unclassified\|Microviridae sp. | / |
|  | SC | IC3_contig_8001 | 3.588 | 0.033 | Podoviridae\|unclassified\|Cellulophaga phage phi18:3 | / |
|  | SC | ID3_contig_983 | 3.420 | 0.026 | Siphoviridae\|unclassified\|Flavobacterium phage vB_FspS_filifjonk9-1 | / |
|  | SC | SC1_contig_2983 | 3.402 | 0.043 | unclassified\|unclassified\|Enterococcus phage MSF1 | / |
|  | SC | IB2_contig_2696 | 3.125 | 0.030 | Microviridae\|unclassified\|Microviridae sp. | / |
|  | SC | SC2_contig_16780 | 3.125 | 0.034 | Microviridae\|unclassified\|Microviridae sp. | / |
|  | SC | ID1_contig_2878 | 3.122 | 0.030 | Microviridae\|unclassified\|Microviridae sp. | / |
|  | SC | IC1_contig_7055 | 3.100 | 0.030 | Microviridae\|unclassified\|Microviridae sp. | / |
|  | SC | SD1_contig_16646 | 3.100 | 0.030 | Microviridae\|unclassified\|Microviridae sp. | / |
|  | SC | IA2_contig_5517 | 3.098 | 0.030 | Microviridae\|unclassified\|Microviridae sp. | / |
|  | SC | SC1_contig_33493 | 3.097 | 0.031 | Microviridae\|unclassified\|Microviridae sp. | / |
|  | SC | IC3_contig_4324 | 3.091 | 0.030 | Microviridae\|unclassified\|Microviridae sp. | / |
|  | SC | IC2_contig_8818 | 3.077 | 0.030 | Microviridae\|unclassified\|Microviridae sp. | / |
|  | SD | SD1_contig_12345 | 4.771 | 0.031 | Microviridae\|unclassified\|Microviridae sp. | / |
|  | SD | SD2_contig_23559 | 3.963 | 0.024 | Microviridae\|unclassified\|Microviridae sp. | / |

LEfSe analysis showed differentially abundant viruses (contig level) among four culture stages in shrimp intestine and sediment based on *P* < 0.05 and a LDA score > 3.0. The viral host was predicted by CRISPR spacer match.

**Table S5 Putative genes detected in identified viral contigs of shrimp intestine and sediment.**

| Level 1 pathway | Level 2 pathway | number of  gene hits |
| --- | --- | --- |
| Organismal Systems | Aging | 5 |
| Organismal Systems | Immune system | 8 |
| Not Included in Pathway or Brite | Unclassified: metabolism | 31 |
| Not Included in Pathway or Brite | Unclassified: genetic information processing | 66 |
| Not Included in Pathway or Brite | Poorly characterized | 45 |
| Not Included in Pathway or Brite | Unclassified: signaling and cellular processes | 36 |
| Metabolism | Carbohydrate metabolism | 22 |
| Metabolism | Energy metabolism | 9 |
| Metabolism | Metabolism of other amino acids | 5 |
| Metabolism | Metabolism of cofactors and vitamins | 77 |
| Metabolism | Nucleotide metabolism | 137 |
| Metabolism | Amino acid metabolism | 63 |
| Metabolism | Lipid metabolism | 1 |
| Metabolism | Xenobiotics biodegradation and metabolism | 1 |
| Metabolism | Metabolism of terpenoids and polyketides | 2 |
| Metabolism | Biosynthesis of other secondary metabolites | 4 |
| Human Diseases | Neurodegenerative disease | 37 |
| Human Diseases | Infectious disease: bacterial | 9 |
| Human Diseases | Drug resistance: antineoplastic | 13 |
| Human Diseases | Cancer: overview | 52 |
| Human Diseases | Endocrine and metabolic disease | 6 |
| Human Diseases | Infectious disease: parasitic | 1 |
| Human Diseases | Cardiovascular disease | 2 |
| Human Diseases | Immune disease | 1 |
| Human Diseases | Drug resistance: antimicrobial | 1 |
| Genetic Information Processing | Translation | 12 |
| Genetic Information Processing | Replication and repair | 151 |
| Genetic Information Processing | Folding, sorting and degradation | 4 |
| Genetic Information Processing | Transcription | 3 |
| Environmental Information Processing | Signal transduction | 13 |
| Environmental Information Processing | Membrane transport | 6 |
| Cellular Processes | Transport and catabolism | 3 |
| Cellular Processes | Cell growth and death | 28 |
| Cellular Processes | Cellular community - prokaryotes | 6 |
| Cellular Processes | Cell motility | 1 |
| Brite Hierarchies | Protein families: genetic information processing | 447 |
| Brite Hierarchies | Protein families: signaling and cellular processes | 239 |
| Brite Hierarchies | Protein families: metabolism | 26 |

The number of unigene hit with KEGG level 1 and level 2 in viral community.

**Table S6** **Correlation test between viral diversity and prokaryotic diversity of shrimp intestine and sediment.**

|  | Intestine procaryotic richness | | Intestine procaryotic Shannon index | | Sediment procaryotic richness | | Sediment procaryotic Shannon index | |
| --- | --- | --- | --- | --- | --- | --- | --- | --- |
|  | R | *p* | R | *p* | R | *p* | R | *p* |
| viral richness (contig) | -0.112 | 0.729 | -0.070 | 0.829 | 0.287 | 0.366 | 0.098 | 0.762 |
| viral Shannon index (contig) | -0.168 | 0.602 | -0.140 | 0.665 | -0.105 | 0.746 | -0.133 | 0.681 |
| phage richness (contig) | -0.133 | 0.681 | -0.154 | 0.633 | 0.189 | 0.557 | 0.105 | 0.746 |
| phage Shannon index (contig) | 0.105 | 0.746 | 0.056 | 0.863 | 0.273 | 0.391 | 0.301 | 0.342 |
| viral richness (species) | -0.175 | 0.587 | -0.231 | 0.471 | 0.224 | 0.484 | 0.091 | 0.779 |
| viral Shannon index (species) | -0.294 | 0.354 | -0.650 | 0.022 | -0.587 | 0.045 | -0.559 | 0.059 |
| phage richness (species) | -0.168 | 0.602 | -0.329 | 0.297 | 0.224 | 0.484 | 0.126 | 0.697 |
| phage Shannon index (species) | -0.210 | 0.513 | -0.517 | 0.085 | -0.378 | 0.226 | -0.294 | 0.354 |

Table showed the relationship between the α-diversity of virus/phage (contig and species level) and prokaryotic (ASV level) community in shrimp intestine and sediment, and the Spearman correlation coefficient was indicated.

**Table S7 The host prediction of virus**

| Sample | Viral contig | Virus | Predicted_host.sp |
| --- | --- | --- | --- |
| Intestine | SD2_contig_5521 | Viruses_unclassified_unclassified_Caudovirales_Myoviridae_Svunavirus_Bacillus virus 1 | *Geobacillus_lituanicus* |
|  | SD2_contig_5521 | Viruses_unclassified_unclassified_Caudovirales_Myoviridae_Svunavirus_Bacillus virus 1 | *Geobacillus_thermocatenulatus* |
|  | SD2_contig_5521 | Viruses_unclassified_unclassified_Caudovirales_Myoviridae_Svunavirus_Bacillus virus 1 | *Geobacillus_zalihae* |
|  | SD2_contig_5521 | Viruses_unclassified_unclassified_Caudovirales_Myoviridae_Svunavirus_Bacillus virus 1 | *Parageobacillus_thermoglucosidasius* |
|  | IB3_contig_5419 | Viruses_unclassified_unclassified_Caudovirales_Myoviridae_unclassified_Rheinheimera phage vB_RspM_Barba29S | *Paracoccus_lutimaris* |
|  | IB3_contig_5419 | Viruses_unclassified_unclassified_Caudovirales_Myoviridae_unclassified_Rheinheimera phage vB_RspM_Barba29S | *Rubellimicrobium_thermophilum* |
|  | IC2_contig_3810 | Viruses_unclassified_unclassified_Caudovirales_Myoviridae_unclassified_Salmonella phage 118970_sal3 | *Shewanella_chilikensis* |
|  | SD2_contig_18435 | Viruses_unclassified_unclassified_Caudovirales_Myoviridae_unclassified_Thermus phage phi OH2 | *Anoxybacillus_geothermalis* |
|  | SD2_contig_18435 | Viruses_unclassified_unclassified_Caudovirales_Myoviridae_unclassified_Thermus phage phi OH2 | *Geobacillus_kaustophilus* |
|  | SD2_contig_18435 | Viruses_unclassified_unclassified_Caudovirales_Myoviridae_unclassified_Thermus phage phi OH2 | *Geobacillus_sp._FW23* |
|  | IA2_contig_2768 | Viruses_unclassified_unclassified_Caudovirales_Myoviridae_unclassified_Thermus phage phi OH2 | *Geobacillus_sp._Y4.1MC1* |
|  | IA2_contig_2768 | Viruses_unclassified_unclassified_Caudovirales_Myoviridae_unclassified_Thermus phage phi OH2 | *Geobacillus_stearothermophilus* |
|  | SD2_contig_18435 | Viruses_unclassified_unclassified_Caudovirales_Myoviridae_unclassified_Thermus phage phi OH2 | *Geobacillus_stearothermophilus* |
|  | SD2_contig_18435 | Viruses_unclassified_unclassified_Caudovirales_Myoviridae_unclassified_Thermus phage phi OH2 | *Geobacillus_thermodenitrificans* |
|  | SD2_contig_18435 | Viruses_unclassified_unclassified_Caudovirales_Myoviridae_unclassified_Thermus phage phi OH2 | *Geobacillus_thermoleovorans* |
|  | SD2_contig_18435 | Viruses_unclassified_unclassified_Caudovirales_Myoviridae_unclassified_Thermus phage phi OH2 | *Parageobacillus_toebii* |
|  | IA2_contig_2104 | Viruses_unclassified_unclassified_Caudovirales_Myoviridae_unclassified_Vibrio phage 1.159.O._10N.261.46.F12 | *Vibrio_metoecus* |
|  | SD3_contig_20479 | Viruses_unclassified_unclassified_Caudovirales_Myoviridae_unclassified_Yersinia phage fEV-1 | *Gemmobacter_caeni* |
|  | IA3_contig_4848 | Viruses_unclassified_unclassified_Caudovirales_Myoviridae_Vhmlvirus_Vibrio virus MAR | *Salinivibrio_sp._DV* |
|  | IA3_contig_2519 | Viruses_unclassified_unclassified_Caudovirales_Myoviridae_Vhmlvirus_Vibrio virus MAR | *Shewanella_algae* |
|  | IA3_contig_2764 | Viruses_unclassified_unclassified_Caudovirales_Myoviridae_Vhmlvirus_Vibrio virus MAR | *Shewanella_algae* |
|  | IA2_contig_1008 | Viruses_unclassified_unclassified_Caudovirales_Myoviridae_Vhmlvirus_Vibrio virus MAR | *Vibrio_gazogenes* |
|  | IA3_contig_1005 | Viruses_unclassified_unclassified_Caudovirales_Myoviridae_Vhmlvirus_Vibrio virus MAR | *Vibrio_cholerae* |
|  | IA3_contig_2519 | Viruses_unclassified_unclassified_Caudovirales_Myoviridae_Vhmlvirus_Vibrio virus MAR | *Vibrio_cholerae* |
|  | IA3_contig_4848 | Viruses_unclassified_unclassified_Caudovirales_Myoviridae_Vhmlvirus_Vibrio virus MAR | *Vibrio_cholerae* |
|  | IA2_contig_4346 | Viruses_unclassified_unclassified_Caudovirales_Myoviridae_Vhmlvirus_Vibrio virus VP585 | *Vibrio_gazogenes* |
|  | IA3_contig_6246 | Viruses_unclassified_unclassified_Caudovirales_Myoviridae_Vhmlvirus_Vibrio virus VP585 | *Vibrio_gazogenes* |
|  | IA1_contig_4598 | Viruses_unclassified_unclassified_Caudovirales_Myoviridae_Vhmlvirus_Vibrio virus VP585 | *Vibrio_cholerae* |
|  | IA2_contig_705 | Viruses_unclassified_unclassified_Caudovirales_Myoviridae_Vhmlvirus_Vibrio virus VP585 | *Vibrio_cholerae* |
|  | IA3_contig_8200 | Viruses_unclassified_unclassified_Caudovirales_Myoviridae_Vhmlvirus_Vibrio virus VP585 | *Vibrio_cholerae* |
|  | SA3_contig_8687 | Viruses_unclassified_unclassified_Caudovirales_Podoviridae_Hollowayvirus_Pseudomonas virus F116 | *Chromobacterium_vaccinii* |
|  | IA3_contig_5507 | Viruses_unclassified_unclassified_Caudovirales_Podoviridae_Hollowayvirus_Pseudomonas virus H66 | *Pseudomonas_aeruginosa* |
|  | ID3_contig_7861 | Viruses_unclassified_unclassified_Caudovirales_Podoviridae_unclassified_Vibrio phage 1.211.A._10N.222.52.F11 | *Salinivibrio_kushneri* |
|  | ID3_contig_7861 | Viruses_unclassified_unclassified_Caudovirales_Podoviridae_unclassified_Vibrio phage 1.211.A._10N.222.52.F11 | *Salinivibrio_sp._MA607* |
|  | ID3_contig_7861 | Viruses_unclassified_unclassified_Caudovirales_Podoviridae_unclassified_Vibrio phage 1.211.A._10N.222.52.F11 | *Shewanella_chilikensis* |
|  | ID3_contig_7861 | Viruses_unclassified_unclassified_Caudovirales_Podoviridae_unclassified_Vibrio phage 1.211.A._10N.222.52.F11 | *Vibrio_gazogenes* |
|  | IA3_contig_1385 | Viruses_unclassified_unclassified_Caudovirales_Siphoviridae_Beetrevirus_Pseudomonas virus PM105 | *Pseudomonas_aeruginosa* |
|  | IC2_contig_2206 | Viruses_unclassified_unclassified_Caudovirales_Siphoviridae_Cornellvirus_Salmonella phage St162 | *Chromohalobacter_salexigens* |
|  | SD1_contig_9362 | Viruses_unclassified_unclassified_Caudovirales_Siphoviridae_Helsingorvirus_Cellulophaga virus Cba121 | *Clostridium_cochlearium* |
|  | ID1_contig_849 | Viruses_unclassified_unclassified_Caudovirales_Siphoviridae_Inhavirus_Nonlabens virus P12024L | *Salinivirga_cyanobacteriivorans* |
|  | IA2_contig_5538 | Viruses_unclassified_unclassified_Caudovirales_Siphoviridae_Jerseyvirus_Salmonella phage St161 | *Acinetobacter_sp._FDAARGOS_515* |
|  | IA2_contig_5538 | Viruses_unclassified_unclassified_Caudovirales_Siphoviridae_Jerseyvirus_Salmonella phage St161 | *Acinetobacter_ursingii* |
|  | SB3_contig_3630 | Viruses_unclassified_unclassified_Caudovirales_Siphoviridae_Phayoncevirus_Mycobacterium virus Phayonce | *Actinomyces_sp._299* |
|  | SB3_contig_3630 | Viruses_unclassified_unclassified_Caudovirales_Siphoviridae_Phayoncevirus_Mycobacterium virus Phayonce | *Propionibacterium_acidifaciens* |
|  | SD2_contig_15762 | Viruses_unclassified_unclassified_Caudovirales_Siphoviridae_Sanovirus_Xylella phage Bacata | *Lysobacter_pythonis* |
|  | IC3_contig_10776 | Viruses_unclassified_unclassified_Caudovirales_Siphoviridae_unclassified_Aeromonas phage AhSzq-1 | *Acinetobacter_baumannii* |
|  | IC2_contig_3326 | Viruses_unclassified_unclassified_Caudovirales_Siphoviridae_unclassified_Enterococcus phage EFC-1 | *Enterococcus_faecalis* |
|  | IC2_contig_5334 | Viruses_unclassified_unclassified_Caudovirales_Siphoviridae_unclassified_Enterococcus phage EFC-1 | *Enterococcus_faecalis* |
|  | IC2_contig_7192 | Viruses_unclassified_unclassified_Caudovirales_Siphoviridae_unclassified_Enterococcus phage EFC-1 | *Enterococcus_faecalis* |
|  | IC2_contig_2806 | Viruses_unclassified_unclassified_Caudovirales_Siphoviridae_unclassified_Enterococcus phage phiEf11 | *Enterococcus_faecalis* |
|  | IC2_contig_4626 | Viruses_unclassified_unclassified_Caudovirales_Siphoviridae_unclassified_Enterococcus phage phiEf11 | *Enterococcus_faecalis* |
|  | IC2_contig_5169 | Viruses_unclassified_unclassified_Caudovirales_Siphoviridae_unclassified_Enterococcus phage phiEf11 | *Enterococcus_faecalis* |
|  | IC2_contig_681 | Viruses_unclassified_unclassified_Caudovirales_Siphoviridae_unclassified_Enterococcus phage vB_EfaS_IME197 | *Enterococcus_faecalis* |
|  | IC2_contig_7200 | Viruses_unclassified_unclassified_Caudovirales_Siphoviridae_unclassified_Enterococcus phage vB_EfaS_IME197 | *Enterococcus_faecalis* |
|  | IC2_contig_7462 | Viruses_unclassified_unclassified_Caudovirales_Siphoviridae_unclassified_Enterococcus phage vB_EfaS_IME197 | *Enterococcus_faecalis* |
|  | IC2_contig_7462 | Viruses_unclassified_unclassified_Caudovirales_Siphoviridae_unclassified_Enterococcus phage vB_EfaS_IME197 | *Enterococcus_sp._1140_ESPC* |
|  | ID2_contig_5686 | Viruses_unclassified_unclassified_Caudovirales_Siphoviridae_unclassified_Flavobacterium phage vB_FspS_snork6-2 | *Flavobacterium_sp._GSN2* |
|  | ID2_contig_5686 | Viruses_unclassified_unclassified_Caudovirales_Siphoviridae_unclassified_Flavobacterium phage vB_FspS_snork6-2 | *Flavobacterium_columnare* |
|  | SC2_contig_5484 | Viruses_unclassified_unclassified_Caudovirales_Siphoviridae_unclassified_Geobacillus virus E2 | *[Bacillus]_caldolyticus* |
|  | SC2_contig_5484 | Viruses_unclassified_unclassified_Caudovirales_Siphoviridae_unclassified_Geobacillus virus E2 | *Anoxybacillus_flavithermus* |
|  | SC2_contig_5484 | Viruses_unclassified_unclassified_Caudovirales_Siphoviridae_unclassified_Geobacillus virus E2 | *Anoxybacillus_geothermalis* |
|  | SC2_contig_5484 | Viruses_unclassified_unclassified_Caudovirales_Siphoviridae_unclassified_Geobacillus virus E2 | *Caldibacillus_debilis* |
|  | SC2_contig_5484 | Viruses_unclassified_unclassified_Caudovirales_Siphoviridae_unclassified_Geobacillus virus E2 | *Geobacillus_sp._1017* |
|  | SC2_contig_5484 | Viruses_unclassified_unclassified_Caudovirales_Siphoviridae_unclassified_Geobacillus virus E2 | *Geobacillus_sp._A8* |
|  | SC2_contig_5484 | Viruses_unclassified_unclassified_Caudovirales_Siphoviridae_unclassified_Geobacillus virus E2 | *Geobacillus_sp._B4113_201601* |
|  | SC2_contig_5484 | Viruses_unclassified_unclassified_Caudovirales_Siphoviridae_unclassified_Geobacillus virus E2 | *Geobacillus_sp._FJAT-46040* |
|  | SC2_contig_5484 | Viruses_unclassified_unclassified_Caudovirales_Siphoviridae_unclassified_Geobacillus virus E2 | *Geobacillus_sp._FW23* |
|  | SC2_contig_5484 | Viruses_unclassified_unclassified_Caudovirales_Siphoviridae_unclassified_Geobacillus virus E2 | *Geobacillus_sp._JS12* |
|  | SC2_contig_5484 | Viruses_unclassified_unclassified_Caudovirales_Siphoviridae_unclassified_Geobacillus virus E2 | *Geobacillus_sp._LYN3* |
|  | SC2_contig_5484 | Viruses_unclassified_unclassified_Caudovirales_Siphoviridae_unclassified_Geobacillus virus E2 | *Geobacillus_sp._T6* |
|  | SC2_contig_5484 | Viruses_unclassified_unclassified_Caudovirales_Siphoviridae_unclassified_Geobacillus virus E2 | *Geobacillus_sp._WSUCF-018B* |
|  | SC2_contig_5484 | Viruses_unclassified_unclassified_Caudovirales_Siphoviridae_unclassified_Geobacillus virus E2 | *Geobacillus_sp._WSUCF1* |
|  | SC2_contig_5484 | Viruses_unclassified_unclassified_Caudovirales_Siphoviridae_unclassified_Geobacillus virus E2 | *Geobacillus_sp._Y412MC52* |
|  | SC2_contig_5484 | Viruses_unclassified_unclassified_Caudovirales_Siphoviridae_unclassified_Geobacillus virus E2 | *Geobacillus_sp._Y412MC61* |
|  | SC2_contig_5484 | Viruses_unclassified_unclassified_Caudovirales_Siphoviridae_unclassified_Geobacillus virus E2 | *Geobacillus_sp._c56-T3* |
|  | SC2_contig_5484 | Viruses_unclassified_unclassified_Caudovirales_Siphoviridae_unclassified_Geobacillus virus E2 | *Geobacillus_stearothermophilus* |
|  | SC2_contig_5484 | Viruses_unclassified_unclassified_Caudovirales_Siphoviridae_unclassified_Geobacillus virus E2 | *Geobacillus_subterraneus* |
|  | SC2_contig_5484 | Viruses_unclassified_unclassified_Caudovirales_Siphoviridae_unclassified_Geobacillus virus E2 | *Geobacillus_thermocatenulatus* |
|  | SC2_contig_5484 | Viruses_unclassified_unclassified_Caudovirales_Siphoviridae_unclassified_Geobacillus virus E2 | *Geobacillus_thermoleovorans* |
|  | SC2_contig_5484 | Viruses_unclassified_unclassified_Caudovirales_Siphoviridae_unclassified_Geobacillus virus E2 | *Geobacillus_zalihae* |
|  | SC2_contig_5484 | Viruses_unclassified_unclassified_Caudovirales_Siphoviridae_unclassified_Geobacillus virus E2 | *Parageobacillus_thermoglucosidasius* |
|  | SC2_contig_5484 | Viruses_unclassified_unclassified_Caudovirales_Siphoviridae_unclassified_Geobacillus virus E2 | *Parageobacillus_toebii* |
|  | SC1_contig_18448 | Viruses_unclassified_unclassified_Caudovirales_Siphoviridae_unclassified_Gordonia phage PhorbesPhlower | *Actinomyces_naeslundii* |
|  | SB2_contig_268 | Viruses_unclassified_unclassified_Caudovirales_Siphoviridae_unclassified_Gordonia phage PhorbesPhlower | *Actinomyces_sp._410* |
|  | SB2_contig_268 | Viruses_unclassified_unclassified_Caudovirales_Siphoviridae_unclassified_Gordonia phage PhorbesPhlower | *Actinomyces_sp._553* |
|  | SB2_contig_268 | Viruses_unclassified_unclassified_Caudovirales_Siphoviridae_unclassified_Gordonia phage PhorbesPhlower | *Kineosphaera_limosa* |
|  | SB2_contig_268 | Viruses_unclassified_unclassified_Caudovirales_Siphoviridae_unclassified_Gordonia phage PhorbesPhlower | *Xylanimicrobium_sp._FW10M-9* |
|  | IB2_contig_11396 | Viruses_unclassified_unclassified_Caudovirales_Siphoviridae_unclassified_Gordonia phage Squiddly | *Gordonia_iterans* |
|  | SD2_contig_21115 | Viruses_unclassified_unclassified_Caudovirales_Siphoviridae_unclassified_Idiomarinaceae phage 1N2-2 | *Thauera_hydrothermalis* |
|  | SD2_contig_22707 | Viruses_unclassified_unclassified_Caudovirales_Siphoviridae_unclassified_Idiomarinaceae phage 1N2-2 | *Zoogloeaceae_bacteirum_Par-f-2* |
|  | IC2_contig_4464 | Viruses_unclassified_unclassified_Caudovirales_Siphoviridae_unclassified_Pseudoalteromonas phage XCL1123 | *Rheinheimera_sp._A13L* |
|  | IA3_contig_4410 | Viruses_unclassified_unclassified_Caudovirales_Siphoviridae_unclassified_Rhizobium phage 16-3 | *Dickeya_sp._FVG10-MFV-A16* |
|  | IA3_contig_4410 | Viruses_unclassified_unclassified_Caudovirales_Siphoviridae_unclassified_Rhizobium phage 16-3 | *Leucothrix_pacifica* |
|  | IA3_contig_4410 | Viruses_unclassified_unclassified_Caudovirales_Siphoviridae_unclassified_Rhizobium phage 16-3 | *Pseudonocardia_sp._Ae717_Ps2* |
|  | IA3_contig_4410 | Viruses_unclassified_unclassified_Caudovirales_Siphoviridae_unclassified_Rhizobium phage 16-3 | *Rubellimicrobium_thermophilum* |
|  | SA1_contig_1411 | Viruses_unclassified_unclassified_Caudovirales_Siphoviridae_unclassified_Rhodococcus phage Jace | *Streptomyces_sp._TSRI0261* |
|  | IC2_contig_294 | Viruses_unclassified_unclassified_Caudovirales_Siphoviridae_unclassified_Salicola phage CGphi29 | *Thauera_hydrothermalis* |
|  | SA2_contig_5230 | Viruses_unclassified_unclassified_Caudovirales_Siphoviridae_unclassified_Streptococcus phage Javan249 | *Serpentinomonas_raichei* |
|  | IA3_contig_5496 | Viruses_unclassified_unclassified_Caudovirales_Siphoviridae_unclassified_Vibrio phage 1.022.O._10N.286.45.A10 | *Vibrio_sp._V11_P1A41T118* |
|  | IA3_contig_4976 | Viruses_unclassified_unclassified_Caudovirales_Siphoviridae_unclassified_Vibrio phage 1.022.O._10N.286.45.A10 | *Vibrio_cholerae* |
|  | IA3_contig_4590 | Viruses_unclassified_unclassified_Caudovirales_Siphoviridae_unclassified_Vibrio phage 1.032.O._10N.261.54.F5 | *Vibrio_cholerae* |
|  | IA3_contig_7625 | Viruses_unclassified_unclassified_Caudovirales_Siphoviridae_unclassified_Vibrio phage 1.046.O._10N.286.52.E3 | *Vibrio_cholerae* |
|  | IA3_contig_9267 | Viruses_unclassified_unclassified_Caudovirales_Siphoviridae_unclassified_Vibrio phage 1.088.O._10N.261.46.A1 | *Vibrio_cholerae* |
|  | IA3_contig_5748 | Viruses_unclassified_unclassified_Caudovirales_Siphoviridae_unclassified_Vibrio phage 1.104.O._10N.286.49.A12 | *Vibrio_sp._2017V-1144* |
|  | IA3_contig_5748 | Viruses_unclassified_unclassified_Caudovirales_Siphoviridae_unclassified_Vibrio phage 1.104.O._10N.286.49.A12 | *Vibrio_cholerae* |
|  | IC3_contig_6467 | Viruses_unclassified_unclassified_Caudovirales_Siphoviridae_unclassified_Vibrio phage 1.282.A._10N.286.54.F8 | *Vibrio_cholerae* |
|  | IA3_contig_3832 | Viruses_unclassified_unclassified_Caudovirales_Siphoviridae_unclassified_Vibrio phage 1.283.C._10N.286.55.A1 | *Vibrio_cholerae* |
|  | IA1_contig_5755 | Viruses_unclassified_unclassified_Caudovirales_Siphoviridae_Vieuvirus_Acinetobacter phage vB_AbaS_TRS1 | *Acinetobacter_baumannii* |
|  | IA1_contig_3066 | Viruses_unclassified_unclassified_Caudovirales_Siphoviridae_Vieuvirus_Acinetobacter phage vB_AbaS_TRS1 | *Acinetobacter_junii* |
|  | IA1_contig_5755 | Viruses_unclassified_unclassified_Caudovirales_Siphoviridae_Vieuvirus_Acinetobacter phage vB_AbaS_TRS1 | *Acinetobacter_sp._A47* |
|  | IA1_contig_5755 | Viruses_unclassified_unclassified_Caudovirales_Siphoviridae_Vieuvirus_Acinetobacter phage vB_AbaS_TRS1 | *Acinetobacter_sp._FDAARGOS_558* |
|  | SB1_contig_7334 | Viruses_unclassified_unclassified_Caudovirales_Siphoviridae_Vieuvirus_Acinetobacter virus B1251 | *Acinetobacter_baumannii* |
|  | SB1_contig_7334 | Viruses_unclassified_unclassified_Caudovirales_Siphoviridae_Vieuvirus_Acinetobacter virus B1251 | *Acinetobacter_parvus* |
|  | IC1_contig_4466 | Viruses_unclassified_unclassified_Herpesvirales_Herpesviridae_Rhadinovirus_Saimiriine gammaherpesvirus 2 | *Bacillus_cereus* |
|  | IC2_contig_4472 | Viruses_unclassified_unclassified_unclassified_Inoviridae_Fibrovirus_Vibrio phage ND1-fs1 | *Vibrio_sp._2014V-1107* |
|  | IC2_contig_4472 | Viruses_unclassified_unclassified_unclassified_Inoviridae_Fibrovirus_Vibrio phage ND1-fs1 | *Vibrio_sp._2016V-1114* |
|  | IC2_contig_1714 | Viruses_unclassified_unclassified_unclassified_Inoviridae_Fibrovirus_Vibrio phage ND1-fs1 | *Vibrio_sp._2017V-1110* |
|  | IC2_contig_4472 | Viruses_unclassified_unclassified_unclassified_Inoviridae_Fibrovirus_Vibrio phage ND1-fs1 | *Vibrio_sp._2017V-1110* |
|  | IC2_contig_1714 | Viruses_unclassified_unclassified_unclassified_Inoviridae_Fibrovirus_Vibrio phage ND1-fs1 | *Vibrio_sp._2017V-1144* |
|  | IC2_contig_1714 | Viruses_unclassified_unclassified_unclassified_Inoviridae_Fibrovirus_Vibrio phage ND1-fs1 | *Vibrio_cholerae* |
|  | IC2_contig_4472 | Viruses_unclassified_unclassified_unclassified_Inoviridae_Fibrovirus_Vibrio phage ND1-fs1 | *Vibrio_cholerae* |
|  | IC2_contig_5512 | Viruses_unclassified_unclassified_unclassified_Inoviridae_Fibrovirus_Vibrio virus VGJ | *Vibrio_sp._2017V-1110* |
|  | IC2_contig_6615 | Viruses_unclassified_unclassified_unclassified_Inoviridae_Fibrovirus_Vibrio virus VGJ | *Vibrio_sp._2017V-1110* |
|  | IC2_contig_5512 | Viruses_unclassified_unclassified_unclassified_Inoviridae_Fibrovirus_Vibrio virus VGJ | *Vibrio_sp._2017V-1144* |
|  | IC2_contig_5512 | Viruses_unclassified_unclassified_unclassified_Inoviridae_Fibrovirus_Vibrio virus VGJ | *Vibrio_cholerae* |
|  | IC2_contig_6615 | Viruses_unclassified_unclassified_unclassified_Inoviridae_Fibrovirus_Vibrio virus VGJ | *Vibrio_cholerae* |
|  | IC2_contig_3964 | Viruses_unclassified_unclassified_unclassified_Inoviridae_Saetivirus_Vibrio virus VFJ | *Vibrio_sp._2016V-1111* |
|  | IC2_contig_3964 | Viruses_unclassified_unclassified_unclassified_Inoviridae_Saetivirus_Vibrio virus VFJ | *Vibrio_sp._2016V-1114* |
|  | IC1_contig_7062 | Viruses_unclassified_unclassified_unclassified_Inoviridae_Saetivirus_Vibrio virus VFJ | *Vibrio_cholerae* |
|  | IC2_contig_3964 | Viruses_unclassified_unclassified_unclassified_Inoviridae_Saetivirus_Vibrio virus VFJ | *Vibrio_cholerae* |
|  | IC1_contig_7070 | Viruses_unclassified_unclassified_unclassified_Inoviridae_unclassified_Vibrio phage K05K4_VK05K4_1 | *Vibrio_metschnikovii* |
|  | IB3_contig_4222 | Viruses_unclassified_unclassified_unclassified_Microviridae_unclassified_Apis mellifera associated microvirus 18 | *Megasphaera_sp._An286* |
|  | IB2_contig_13314 | Viruses_unclassified_unclassified_unclassified_Microviridae_unclassified_Apis mellifera associated microvirus 4 | *Tyzzerella_nexilis* |
|  | IB1_contig_1993 | Viruses_unclassified_unclassified_unclassified_Microviridae_unclassified_Gokushovirus WZ-2015a | *Burkholderiales_bacterium* |
|  | IB1_contig_5576 | Viruses_unclassified_unclassified_unclassified_Microviridae_unclassified_Gokushovirus WZ-2015a | *Burkholderiales_bacterium* |
|  | IB1_contig_6456 | Viruses_unclassified_unclassified_unclassified_Microviridae_unclassified_Gokushovirus WZ-2015a | *Burkholderiales_bacterium* |
|  | IB1_contig_17237 | Viruses_unclassified_unclassified_unclassified_Microviridae_unclassified_Gokushovirus WZ-2015a | *Clostridiales_bacterium_VE202-03* |
|  | IB1_contig_17237 | Viruses_unclassified_unclassified_unclassified_Microviridae_unclassified_Gokushovirus WZ-2015a | *Clostridium_sp._ATCC_BAA-442* |
|  | IB1_contig_3736 | Viruses_unclassified_unclassified_unclassified_Microviridae_unclassified_Gokushovirus WZ-2015a | *Enterobacter_cancerogenus* |
|  | IB1_contig_15519 | Viruses_unclassified_unclassified_unclassified_Microviridae_unclassified_Gokushovirus WZ-2015a | *Faecalibacterium_prausnitzii* |
|  | IB1_contig_16710 | Viruses_unclassified_unclassified_unclassified_Microviridae_unclassified_Gokushovirus WZ-2015a | *Faecalibacterium_sp._OM04-11BH* |
|  | IB1_contig_17237 | Viruses_unclassified_unclassified_unclassified_Microviridae_unclassified_Gokushovirus WZ-2015a | *Flavonifractor_plautii* |
|  | IB1_contig_6420 | Viruses_unclassified_unclassified_unclassified_Microviridae_unclassified_Gokushovirus WZ-2015a | *Phascolarctobacterium_faecium* |
|  | IB1_contig_9235 | Viruses_unclassified_unclassified_unclassified_Microviridae_unclassified_Gokushovirus WZ-2015a | *Phascolarctobacterium_faecium* |
|  | IB1_contig_3736 | Viruses_unclassified_unclassified_unclassified_Microviridae_unclassified_Gokushovirus WZ-2015a | *Prosthecochloris_sp._ZM_2* |
|  | IB1_contig_1175 | Viruses_unclassified_unclassified_unclassified_Microviridae_unclassified_Human gut gokushovirus | *Faecalibacterium_prausnitzii* |
|  | IB1_contig_411 | Viruses_unclassified_unclassified_unclassified_Microviridae_unclassified_Human gut gokushovirus | *Faecalibacterium_prausnitzii* |
|  | IB1_contig_11963 | Viruses_unclassified_unclassified_unclassified_Microviridae_unclassified_Human gut gokushovirus | *Phascolarctobacterium_faecium* |
|  | IB1_contig_12561 | Viruses_unclassified_unclassified_unclassified_Microviridae_unclassified_Human gut gokushovirus | *Phascolarctobacterium_faecium* |
|  | IB1_contig_17026 | Viruses_unclassified_unclassified_unclassified_Microviridae_unclassified_Human gut gokushovirus | *Phascolarctobacterium_faecium* |
|  | ID3_contig_7729 | Viruses_unclassified_unclassified_unclassified_Microviridae_unclassified_Microviridae sp. | *Bathymodiolus_thermophilus_thioautotrophic_gill_symbiont* |
|  | SB3_contig_14568 | Viruses_unclassified_unclassified_unclassified_Microviridae_unclassified_Microviridae sp. | *Candidimonas_nitroreducens* |
|  | IA3_contig_10563 | Viruses_unclassified_unclassified_unclassified_Microviridae_unclassified_Microviridae sp. | *Enterobacter_kobei* |
|  | IB3_contig_1522 | Viruses_unclassified_unclassified_unclassified_Microviridae_unclassified_Microviridae sp. | *Haliscomenobacter_hydrossis* |
|  | IB2_contig_3254 | Viruses_unclassified_unclassified_unclassified_Microviridae_unclassified_Microviridae sp. | *Helicobacter_muridarum* |
|  | IA1_contig_2635 | Viruses_unclassified_unclassified_unclassified_Microviridae_unclassified_Microviridae sp. | *Legionella_pneumophila* |
|  | SB1_contig_14011 | Viruses_unclassified_unclassified_unclassified_Microviridae_unclassified_Microviridae sp. | *Legionella_pneumophila* |
|  | SB3_contig_14568 | Viruses_unclassified_unclassified_unclassified_Microviridae_unclassified_Microviridae sp. | *Legionella_pneumophila* |
|  | SD3_contig_1859 | Viruses_unclassified_unclassified_unclassified_Microviridae_unclassified_Microviridae sp. | *Legionella_pneumophila* |
|  | IB1_contig_17388 | Viruses_unclassified_unclassified_unclassified_Microviridae_unclassified_Microviridae sp. | *Marinomonas_arctica* |
|  | IC3_contig_11759 | Viruses_unclassified_unclassified_unclassified_Microviridae_unclassified_Microviridae sp. | *Prosthecochloris_sp._ZM_2* |
|  | SC1_contig_33566 | Viruses_unclassified_unclassified_unclassified_Microviridae_unclassified_Microviridae sp. | *Salmonella_enterica* |
|  | IB1_contig_4210 | Viruses_unclassified_unclassified_unclassified_Microviridae_unclassified_Tortoise microvirus 13 | *Bacteroides_sp._HMSC068A09* |
|  | IC3_contig_1612 | Viruses_unclassified_unclassified_unclassified_Microviridae_unclassified_Tortoise microvirus 38 | *Paracoccus_pantotrophus* |
|  | IB1_contig_8709 | Viruses_unclassified_unclassified_unclassified_Microviridae_unclassified_Tortoise microvirus 40 | *Bacteroides_dorei* |
|  | IB1_contig_8709 | Viruses_unclassified_unclassified_unclassified_Microviridae_unclassified_Tortoise microvirus 40 | *Bacteroides_vulgatus* |
|  | SD1_contig_9068 | Viruses_unclassified_unclassified_unclassified_unclassified_unclassified_Marine virus AFVG_117M46 | *Salinispora_arenicola* |
|  | IB3_contig_6080 | Viruses_unclassified_unclassified_unclassified_unclassified_unclassified_Marine virus AFVG_25M68 | *Streptococcus_oralis* |
|  | SD2_contig_1103 | Viruses_unclassified_unclassified_unclassified_unclassified_unclassified_Prokaryotic dsDNA virus sp. | *Chromobacterium_haemolyticum* |
|  | SD3_contig_14231 | Viruses_unclassified_unclassified_unclassified_unclassified_unclassified_Prokaryotic dsDNA virus sp. | *Lacinutrix_sp._Bg11-31* |
|  | SB3_contig_897 | Viruses_unclassified_unclassified_unclassified_unclassified_unclassified_Prokaryotic dsDNA virus sp. | *Porphyromonas_gingivalis* |
|  | IA3_contig_2938 | Viruses_unclassified_unclassified_unclassified_unclassified_unclassified_Prokaryotic dsDNA virus sp. | *Pseudoalteromonas_flavipulchra* |
| Sediment | SA1_contig_11168 | Viruses_unclassified_unclassified_Caudovirales_Myoviridae_Obolenskvirus_Acinetobacter virus AbP2 | *Moraxella_osloensis* |
|  | SD3_contig_26798 | Viruses_unclassified_unclassified_Caudovirales_Myoviridae_Svunavirus_Bacillus virus 1 | *[Bacillus]_caldolyticus* |
|  | SD2_contig_5521 | Viruses_unclassified_unclassified_Caudovirales_Myoviridae_Svunavirus_Bacillus virus 1 | *Geobacillus_lituanicus* |
|  | SD3_contig_26798 | Viruses_unclassified_unclassified_Caudovirales_Myoviridae_Svunavirus_Bacillus virus 1 | *Geobacillus_stearothermophilus* |
|  | SD3_contig_26798 | Viruses_unclassified_unclassified_Caudovirales_Myoviridae_Svunavirus_Bacillus virus 1 | *Geobacillus_subterraneus* |
|  | SD2_contig_5521 | Viruses_unclassified_unclassified_Caudovirales_Myoviridae_Svunavirus_Bacillus virus 1 | *Geobacillus_thermocatenulatus* |
|  | SB3_contig_14174 | Viruses_unclassified_unclassified_Caudovirales_Myoviridae_Svunavirus_Bacillus virus 1 | *Geobacillus_thermoleovorans* |
|  | SD3_contig_26798 | Viruses_unclassified_unclassified_Caudovirales_Myoviridae_Svunavirus_Bacillus virus 1 | *Geobacillus_thermoleovorans* |
|  | SD3_contig_26798 | Viruses_unclassified_unclassified_Caudovirales_Myoviridae_Svunavirus_Bacillus virus 1 | *Geobacillus_uzenensis* |
|  | SD2_contig_5521 | Viruses_unclassified_unclassified_Caudovirales_Myoviridae_Svunavirus_Bacillus virus 1 | *Geobacillus_zalihae* |
|  | SD2_contig_5521 | Viruses_unclassified_unclassified_Caudovirales_Myoviridae_Svunavirus_Bacillus virus 1 | *Parageobacillus_thermoglucosidasius* |
|  | SA1_contig_6678 | Viruses_unclassified_unclassified_Caudovirales_Myoviridae_unclassified_Proteus phage PM2 | *Moraxella_osloensis* |
|  | IB3_contig_5419 | Viruses_unclassified_unclassified_Caudovirales_Myoviridae_unclassified_Rheinheimera phage vB_RspM_Barba29S | *Paracoccus_lutimaris* |
|  | IB3_contig_5419 | Viruses_unclassified_unclassified_Caudovirales_Myoviridae_unclassified_Rheinheimera phage vB_RspM_Barba29S | *Rubellimicrobium_thermophilum* |
|  | SD2_contig_18435 | Viruses_unclassified_unclassified_Caudovirales_Myoviridae_unclassified_Thermus phage phi OH2 | *Anoxybacillus_geothermalis* |
|  | SD2_contig_18435 | Viruses_unclassified_unclassified_Caudovirales_Myoviridae_unclassified_Thermus phage phi OH2 | *Geobacillus_kaustophilus* |
|  | SD2_contig_18435 | Viruses_unclassified_unclassified_Caudovirales_Myoviridae_unclassified_Thermus phage phi OH2 | *Geobacillus_sp._FW23* |
|  | SD2_contig_18435 | Viruses_unclassified_unclassified_Caudovirales_Myoviridae_unclassified_Thermus phage phi OH2 | *Geobacillus_stearothermophilus* |
|  | SD2_contig_18435 | Viruses_unclassified_unclassified_Caudovirales_Myoviridae_unclassified_Thermus phage phi OH2 | *Geobacillus_thermodenitrificans* |
|  | SD2_contig_18435 | Viruses_unclassified_unclassified_Caudovirales_Myoviridae_unclassified_Thermus phage phi OH2 | *Geobacillus_thermoleovorans* |
|  | SD2_contig_18435 | Viruses_unclassified_unclassified_Caudovirales_Myoviridae_unclassified_Thermus phage phi OH2 | *Parageobacillus_toebii* |
|  | SD3_contig_20479 | Viruses_unclassified_unclassified_Caudovirales_Myoviridae_unclassified_Yersinia phage fEV-1 | *Gemmobacter_caeni* |
|  | SA3_contig_8687 | Viruses_unclassified_unclassified_Caudovirales_Podoviridae_Hollowayvirus_Pseudomonas virus F116 | *Chromobacterium_vaccinii* |
|  | SA2_contig_4818 | Viruses_unclassified_unclassified_Caudovirales_Podoviridae_Lederbergvirus_Salmonella phage ST160 | *Acidiferrobacter_thiooxydans* |
|  | SD2_contig_12131 | Viruses_unclassified_unclassified_Caudovirales_Siphoviridae_Cheoctovirus_Mycobacterium phage SiSi | *Mycobacterium_persicum* |
|  | SD1_contig_9362 | Viruses_unclassified_unclassified_Caudovirales_Siphoviridae_Helsingorvirus_Cellulophaga virus Cba121 | *Clostridium_cochlearium* |
|  | ID1_contig_849 | Viruses_unclassified_unclassified_Caudovirales_Siphoviridae_Inhavirus_Nonlabens virus P12024L | *Salinivirga_cyanobacteriivorans* |
|  | IA2_contig_5538 | Viruses_unclassified_unclassified_Caudovirales_Siphoviridae_Jerseyvirus_Salmonella phage St161 | *Acinetobacter_sp._FDAARGOS_515* |
|  | IA2_contig_5538 | Viruses_unclassified_unclassified_Caudovirales_Siphoviridae_Jerseyvirus_Salmonella phage St161 | *Acinetobacter_ursingii* |
|  | SC1_contig_20035 | Viruses_unclassified_unclassified_Caudovirales_Siphoviridae_Moineauvirus_Streptococcus phage Javan392 | *Planifilum_fulgidum* |
|  | SB1_contig_5429 | Viruses_unclassified_unclassified_Caudovirales_Siphoviridae_Omegavirus_Mycobacterium virus Omega | *Cellulomonas_iranensis* |
|  | SC2_contig_11717 | Viruses_unclassified_unclassified_Caudovirales_Siphoviridae_Pahexavirus_Cutibacterium phage P108C | *Cutibacterium_avidum* |
|  | SC2_contig_16561 | Viruses_unclassified_unclassified_Caudovirales_Siphoviridae_Pahexavirus_Propionibacterium phage pa63 | *Cutibacterium_acnes* |
|  | SC2_contig_9860 | Viruses_unclassified_unclassified_Caudovirales_Siphoviridae_Pahexavirus_Propionibacterium phage PacnesP1 | *Cutibacterium_acnes* |
|  | SC2_contig_1890 | Viruses_unclassified_unclassified_Caudovirales_Siphoviridae_Pahexavirus_Propionibacterium virus MrAK | *Cutibacterium_avidum* |
|  | SB3_contig_3630 | Viruses_unclassified_unclassified_Caudovirales_Siphoviridae_Phayoncevirus_Mycobacterium virus Phayonce | *Actinomyces_sp._299* |
|  | SB3_contig_3630 | Viruses_unclassified_unclassified_Caudovirales_Siphoviridae_Phayoncevirus_Mycobacterium virus Phayonce | *Propionibacterium_acidifaciens* |
|  | SC2_contig_5625 | Viruses_unclassified_unclassified_Caudovirales_Siphoviridae_Pulverervirus_Propionibacterium phage PFR2 | *Kocuria_varians* |
|  | SD2_contig_15762 | Viruses_unclassified_unclassified_Caudovirales_Siphoviridae_Sanovirus_Xylella phage Bacata | *Lysobacter_pythonis* |
|  | IC3_contig_10776 | Viruses_unclassified_unclassified_Caudovirales_Siphoviridae_unclassified_Aeromonas phage AhSzq-1 | *Acinetobacter_baumannii* |
|  | SD2_contig_3431 | Viruses_unclassified_unclassified_Caudovirales_Siphoviridae_unclassified_Bacillus phage vB_BcoS-136 | *Virgibacillus_dakarensis* |
|  | SC3_contig_4572 | Viruses_unclassified_unclassified_Caudovirales_Siphoviridae_unclassified_Bacteriophage Eos | *Escherichia_coli* |
|  | SA3_contig_7812 | Viruses_unclassified_unclassified_Caudovirales_Siphoviridae_unclassified_Dinoroseobacter phage vB_DshS-R4C | *Pseudomonas_aeruginosa* |
|  | ID2_contig_5686 | Viruses_unclassified_unclassified_Caudovirales_Siphoviridae_unclassified_Flavobacterium phage vB_FspS_snork6-2 | *Flavobacterium_columnare* |
|  | ID2_contig_5686 | Viruses_unclassified_unclassified_Caudovirales_Siphoviridae_unclassified_Flavobacterium phage vB_FspS_snork6-2 | *Flavobacterium_sp._GSN2* |
|  | SC2_contig_5484 | Viruses_unclassified_unclassified_Caudovirales_Siphoviridae_unclassified_Geobacillus virus E2 | *[Bacillus]_caldolyticus* |
|  | SC2_contig_5484 | Viruses_unclassified_unclassified_Caudovirales_Siphoviridae_unclassified_Geobacillus virus E2 | *Anoxybacillus_flavithermus* |
|  | SC2_contig_5484 | Viruses_unclassified_unclassified_Caudovirales_Siphoviridae_unclassified_Geobacillus virus E2 | *Anoxybacillus_geothermalis* |
|  | SC2_contig_5484 | Viruses_unclassified_unclassified_Caudovirales_Siphoviridae_unclassified_Geobacillus virus E2 | *Caldibacillus_debilis* |
|  | SC2_contig_5484 | Viruses_unclassified_unclassified_Caudovirales_Siphoviridae_unclassified_Geobacillus virus E2 | *Geobacillus_sp._1017* |
|  | SC2_contig_5484 | Viruses_unclassified_unclassified_Caudovirales_Siphoviridae_unclassified_Geobacillus virus E2 | *Geobacillus_sp._A8* |
|  | SC2_contig_5484 | Viruses_unclassified_unclassified_Caudovirales_Siphoviridae_unclassified_Geobacillus virus E2 | *Geobacillus_sp._B4113_201601* |
|  | SC2_contig_5484 | Viruses_unclassified_unclassified_Caudovirales_Siphoviridae_unclassified_Geobacillus virus E2 | *Geobacillus_sp._C56-T3* |
|  | SC2_contig_5484 | Viruses_unclassified_unclassified_Caudovirales_Siphoviridae_unclassified_Geobacillus virus E2 | *Geobacillus_sp._FJAT-46040* |
|  | SC2_contig_5484 | Viruses_unclassified_unclassified_Caudovirales_Siphoviridae_unclassified_Geobacillus virus E2 | *Geobacillus_sp._FW23* |
|  | SC2_contig_5484 | Viruses_unclassified_unclassified_Caudovirales_Siphoviridae_unclassified_Geobacillus virus E2 | *Geobacillus_sp._JS12* |
|  | SC2_contig_5484 | Viruses_unclassified_unclassified_Caudovirales_Siphoviridae_unclassified_Geobacillus virus E2 | *Geobacillus_sp._LYN3* |
|  | SC2_contig_5484 | Viruses_unclassified_unclassified_Caudovirales_Siphoviridae_unclassified_Geobacillus virus E2 | *Geobacillus_sp._T6* |
|  | SC2_contig_5484 | Viruses_unclassified_unclassified_Caudovirales_Siphoviridae_unclassified_Geobacillus virus E2 | *Geobacillus_sp._WSUCF-018B* |
|  | SC2_contig_5484 | Viruses_unclassified_unclassified_Caudovirales_Siphoviridae_unclassified_Geobacillus virus E2 | *Geobacillus_sp._WSUCF1* |
|  | SC2_contig_5484 | Viruses_unclassified_unclassified_Caudovirales_Siphoviridae_unclassified_Geobacillus virus E2 | *Geobacillus_sp._Y412MC52* |
|  | SC2_contig_5484 | Viruses_unclassified_unclassified_Caudovirales_Siphoviridae_unclassified_Geobacillus virus E2 | *Geobacillus_sp._Y412MC61* |
|  | SC2_contig_5484 | Viruses_unclassified_unclassified_Caudovirales_Siphoviridae_unclassified_Geobacillus virus E2 | *Geobacillus_stearothermophilus* |
|  | SC2_contig_5484 | Viruses_unclassified_unclassified_Caudovirales_Siphoviridae_unclassified_Geobacillus virus E2 | *Geobacillus_subterraneus* |
|  | SC2_contig_5484 | Viruses_unclassified_unclassified_Caudovirales_Siphoviridae_unclassified_Geobacillus virus E2 | *Geobacillus_thermocatenulatus* |
|  | SC2_contig_5484 | Viruses_unclassified_unclassified_Caudovirales_Siphoviridae_unclassified_Geobacillus virus E2 | *Geobacillus_thermoleovorans* |
|  | SC2_contig_5484 | Viruses_unclassified_unclassified_Caudovirales_Siphoviridae_unclassified_Geobacillus virus E2 | *Geobacillus_zalihae* |
|  | SC2_contig_5484 | Viruses_unclassified_unclassified_Caudovirales_Siphoviridae_unclassified_Geobacillus virus E2 | *Parageobacillus_thermoglucosidasius* |
|  | SC2_contig_5484 | Viruses_unclassified_unclassified_Caudovirales_Siphoviridae_unclassified_Geobacillus virus E2 | *Parageobacillus_toebii* |
|  | SC1_contig_18448 | Viruses_unclassified_unclassified_Caudovirales_Siphoviridae_unclassified_Gordonia phage PhorbesPhlower | *Actinomyces_naeslundii* |
|  | SB2_contig_268 | Viruses_unclassified_unclassified_Caudovirales_Siphoviridae_unclassified_Gordonia phage PhorbesPhlower | *Actinomyces_sp._410* |
|  | SB2_contig_268 | Viruses_unclassified_unclassified_Caudovirales_Siphoviridae_unclassified_Gordonia phage PhorbesPhlower | *Actinomyces_sp._553* |
|  | SB2_contig_268 | Viruses_unclassified_unclassified_Caudovirales_Siphoviridae_unclassified_Gordonia phage PhorbesPhlower | *Kineosphaera_limosa* |
|  | SB2_contig_268 | Viruses_unclassified_unclassified_Caudovirales_Siphoviridae_unclassified_Gordonia phage PhorbesPhlower | *Xylanimicrobium_sp._FW10M-9* |
|  | IB2_contig_11396 | Viruses_unclassified_unclassified_Caudovirales_Siphoviridae_unclassified_Gordonia phage Squiddly | *Gordonia_iterans* |
|  | SD2_contig_21115 | Viruses_unclassified_unclassified_Caudovirales_Siphoviridae_unclassified_Idiomarinaceae phage 1N2-2 | *Thauera_hydrothermalis* |
|  | SD2_contig_22707 | Viruses_unclassified_unclassified_Caudovirales_Siphoviridae_unclassified_Idiomarinaceae phage 1N2-2 | *Zoogloeaceae_bacteirum_Par-f-2* |
|  | SD2_contig_13209 | Viruses_unclassified_unclassified_Caudovirales_Siphoviridae_unclassified_Microbacterium phage Zanella | *Actinomyces_sp._ICM39* |
|  | SA1_contig_13242 | Viruses_unclassified_unclassified_Caudovirales_Siphoviridae_unclassified_Moraxella phage Mcat8 | *Moraxella_osloensis* |
|  | SA3_contig_8446 | Viruses_unclassified_unclassified_Caudovirales_Siphoviridae_unclassified_Mycobacterium phage Sparky | *Actinomyces_sp._oral_taxon_897* |
|  | SC1_contig_26068 | Viruses_unclassified_unclassified_Caudovirales_Siphoviridae_unclassified_Paracoccus phage vB_PbeS_Pben1 | *Gemmobacter_sp._LW-1* |
|  | SD3_contig_16477 | Viruses_unclassified_unclassified_Caudovirales_Siphoviridae_unclassified_Paracoccus phage vB_PbeS_Pben1 | *Paracoccus_lutimaris* |
|  | SA3_contig_6598 | Viruses_unclassified_unclassified_Caudovirales_Siphoviridae_unclassified_Pseudoalteromonas phage TW1 | *Pseudoalteromonas_sp._A757* |
|  | IC2_contig_4464 | Viruses_unclassified_unclassified_Caudovirales_Siphoviridae_unclassified_Pseudoalteromonas phage XCL1123 | *Rheinheimera_sp._A13L* |
|  | SC2_contig_9229 | Viruses_unclassified_unclassified_Caudovirales_Siphoviridae_unclassified_Pseudomonas phage YMC11/02/R656 | *Thiomonas_sp._FB-Cd* |
|  | IA3_contig_4410 | Viruses_unclassified_unclassified_Caudovirales_Siphoviridae_unclassified_Rhizobium phage 16-3 | *Dickeya_sp._FVG10-MFV-A16* |
|  | IA3_contig_4410 | Viruses_unclassified_unclassified_Caudovirales_Siphoviridae_unclassified_Rhizobium phage 16-3 | *Leucothrix_pacifica* |
|  | IA3_contig_4410 | Viruses_unclassified_unclassified_Caudovirales_Siphoviridae_unclassified_Rhizobium phage 16-3 | *Pseudonocardia_sp._Ae717_Ps2* |
|  | IA3_contig_4410 | Viruses_unclassified_unclassified_Caudovirales_Siphoviridae_unclassified_Rhizobium phage 16-3 | *Rubellimicrobium_thermophilum* |
|  | SA1_contig_1411 | Viruses_unclassified_unclassified_Caudovirales_Siphoviridae_unclassified_Rhodococcus phage Jace | *Streptomyces_sp._TSRI0261* |
|  | IC2_contig_294 | Viruses_unclassified_unclassified_Caudovirales_Siphoviridae_unclassified_Salicola phage CGphi29 | *Thauera_hydrothermalis* |
|  | SA2_contig_5230 | Viruses_unclassified_unclassified_Caudovirales_Siphoviridae_unclassified_Streptococcus phage Javan249 | *Serpentinomonas_raichei* |
|  | SC1_contig_21745 | Viruses_unclassified_unclassified_Caudovirales_Siphoviridae_unclassified_Streptococcus phage phi-SsUD.1 | *Coprobacillus_sp._AF36-10BH* |
|  | SC1_contig_5274 | Viruses_unclassified_unclassified_Caudovirales_Siphoviridae_unclassified_Streptomyces phage ToastyFinz | *Kitasatospora_phosalacinea* |
|  | SC2_contig_1581 | Viruses_unclassified_unclassified_Caudovirales_Siphoviridae_unclassified_Synechococcus phage S-CBS3 | *Thauera_sp._27* |
|  | SC3_contig_21391 | Viruses_unclassified_unclassified_Caudovirales_Siphoviridae_unclassified_Tsukamurella phage TPA4 | *Streptomyces_sp._Root1295* |
|  | SC3_contig_21391 | Viruses_unclassified_unclassified_Caudovirales_Siphoviridae_unclassified_Tsukamurella phage TPA4 | *Streptomyces_sp._Root63* |
|  | SA3_contig_8323 | Viruses_unclassified_unclassified_Caudovirales_Siphoviridae_Vieuvirus_Acinetobacter phage Ab105-2phi | *Acinetobacter_baumannii* |
|  | SA3_contig_951 | Viruses_unclassified_unclassified_Caudovirales_Siphoviridae_Vieuvirus_Acinetobacter phage Ab105-2phi | *Acinetobacter_baumannii* |
|  | SB1_contig_8123 | Viruses_unclassified_unclassified_Caudovirales_Siphoviridae_Vieuvirus_Acinetobacter phage Ab105-2phi | *Acinetobacter_baumannii* |
|  | SD2_contig_13972 | Viruses_unclassified_unclassified_Caudovirales_Siphoviridae_Vieuvirus_Acinetobacter phage Ab105-3phi | *Acinetobacter_baumannii* |
|  | SA3_contig_3975 | Viruses_unclassified_unclassified_Caudovirales_Siphoviridae_Vieuvirus_Acinetobacter phage AM106 | *Yersinia_pestis* |
|  | SA3_contig_5655 | Viruses_unclassified_unclassified_Caudovirales_Siphoviridae_Vieuvirus_Acinetobacter phage vB_AbaS_TRS1 | *Acinetobacter_baumannii* |
|  | SD3_contig_24314 | Viruses_unclassified_unclassified_Caudovirales_Siphoviridae_Vieuvirus_Acinetobacter phage vB_AbaS_TRS1 | *Acinetobacter_baumannii* |
|  | SB1_contig_7334 | Viruses_unclassified_unclassified_Caudovirales_Siphoviridae_Vieuvirus_Acinetobacter virus B1251 | *Acinetobacter_baumannii* |
|  | SB1_contig_7334 | Viruses_unclassified_unclassified_Caudovirales_Siphoviridae_Vieuvirus_Acinetobacter virus B1251 | *Acinetobacter_parvus* |
|  | SD3_contig_32285 | Viruses_unclassified_unclassified_Caudovirales_Siphoviridae_Vieuvirus_Acinetobacter virus R3177 | *Acinetobacter_baumannii* |
|  | SD3_contig_9693 | Viruses_unclassified_unclassified_Caudovirales_Siphoviridae_Vieuvirus_Acinetobacter virus R3177 | *Acinetobacter_baumannii* |
|  | SD3_contig_9693 | Viruses_unclassified_unclassified_Caudovirales_Siphoviridae_Vieuvirus_Acinetobacter virus R3177 | *Acinetobacter_pittii* |
|  | SA2_contig_10843 | Viruses_unclassified_unclassified_Caudovirales_Siphoviridae_Wbetavirus_Bacillus phage Negev_SA | *Brevibacillus_laterosporus* |
|  | IC1_contig_4466 | Viruses_unclassified_unclassified_Herpesvirales_Herpesviridae_Rhadinovirus_Saimiriine gammaherpesvirus 2 | *Bacillus_cereus* |
|  | IC1_contig_7062 | Viruses_unclassified_unclassified_unclassified_Inoviridae_Saetivirus_Vibrio virus VFJ | *Vibrio_cholerae* |
|  | IC2_contig_3964 | Viruses_unclassified_unclassified_unclassified_Inoviridae_Saetivirus_Vibrio virus VFJ | *Vibrio_cholerae* |
|  | IC2_contig_3964 | Viruses_unclassified_unclassified_unclassified_Inoviridae_Saetivirus_Vibrio virus VFJ | *Vibrio_sp._2016V-1111* |
|  | IC2_contig_3964 | Viruses_unclassified_unclassified_unclassified_Inoviridae_Saetivirus_Vibrio virus VFJ | *Vibrio_sp._2016V-1114* |
|  | SB1_contig_7311 | Viruses_unclassified_unclassified_unclassified_Inoviridae_unclassified_Inoviridae sp. | *Acinetobacter_parvus* |
|  | SB1_contig_7311 | Viruses_unclassified_unclassified_unclassified_Inoviridae_unclassified_Inoviridae sp. | *Acinetobacter_soli* |
|  | SB1_contig_7311 | Viruses_unclassified_unclassified_unclassified_Inoviridae_unclassified_Inoviridae sp. | *Acinetobacter_sp._CIP_102529* |
|  | SB1_contig_7311 | Viruses_unclassified_unclassified_unclassified_Inoviridae_unclassified_Inoviridae sp. | *Acinetobacter_sp._CIP_102637* |
|  | IC1_contig_7070 | Viruses_unclassified_unclassified_unclassified_Inoviridae_unclassified_Vibrio phage K05K4_VK05K4_1 | *Vibrio_metschnikovii* |
|  | ID3_contig_7729 | Viruses_unclassified_unclassified_unclassified_Microviridae_unclassified_Microviridae sp. | *Bathymodiolus_thermophilus_thioautotrophic_gill_symbiont* |
|  | SD2_contig_23711 | Viruses_unclassified_unclassified_unclassified_Microviridae_unclassified_Microviridae sp. | *Burkholderiales_bacterium_YL45* |
|  | SB3_contig_14568 | Viruses_unclassified_unclassified_unclassified_Microviridae_unclassified_Microviridae sp. | *Candidimonas_nitroreducens* |
|  | IB2_contig_3254 | Viruses_unclassified_unclassified_unclassified_Microviridae_unclassified_Microviridae sp. | *Helicobacter_muridarum* |
|  | SB1_contig_14011 | Viruses_unclassified_unclassified_unclassified_Microviridae_unclassified_Microviridae sp. | *Legionella_pneumophila* |
|  | SB3_contig_14568 | Viruses_unclassified_unclassified_unclassified_Microviridae_unclassified_Microviridae sp. | *Legionella_pneumophila* |
|  | SD3_contig_1859 | Viruses_unclassified_unclassified_unclassified_Microviridae_unclassified_Microviridae sp. | *Legionella_pneumophila* |
|  | SC3_contig_30138 | Viruses_unclassified_unclassified_unclassified_Microviridae_unclassified_Microviridae sp. | *Nitrosospira_multiformis* |
|  | SC3_contig_30138 | Viruses_unclassified_unclassified_unclassified_Microviridae_unclassified_Microviridae sp. | *Nitrosospira_sp._Nsp5* |
|  | SA3_contig_11585 | Viruses_unclassified_unclassified_unclassified_Microviridae_unclassified_Microviridae sp. | *Oxalobacter_formigenes* |
|  | IC3_contig_11759 | Viruses_unclassified_unclassified_unclassified_Microviridae_unclassified_Microviridae sp. | *Prosthecochloris_sp._ZM_2* |
|  | SC1_contig_33566 | Viruses_unclassified_unclassified_unclassified_Microviridae_unclassified_Microviridae sp. | *Salmonella_enterica* |
|  | SD2_contig_23711 | Viruses_unclassified_unclassified_unclassified_Microviridae_unclassified_Microviridae sp. | *Turicimonas_muris* |
|  | IC3_contig_1612 | Viruses_unclassified_unclassified_unclassified_Microviridae_unclassified_Tortoise microvirus 38 | *Paracoccus_pantotrophus* |
|  | SD2_contig_15787 | Viruses_unclassified_unclassified_unclassified_Pithoviridae_unclassified_Pithovirus LCPAC404 | *Clostridioides_difficile* |
|  | SB3_contig_2300 | Viruses_unclassified_unclassified_unclassified_Pithoviridae_unclassified_Solumvirus sp. | *Aneurinibacillus_migulanus* |
|  | SC1_contig_18739 | Viruses_unclassified_unclassified_unclassified_unclassified_Pandoravirus_Pandoravirus neocaledonia | *Mycobacterium_sp._1554424.7* |
|  | SD1_contig_9068 | Viruses_unclassified_unclassified_unclassified_unclassified_unclassified_Marine virus AFVG_117M46 | *Salinispora_arenicola* |
|  | SD2_contig_6783 | Viruses_unclassified_unclassified_unclassified_unclassified_unclassified_Marine virus AFVG_25M296 | *Bacillus_thuringiensis* |
|  | IB3_contig_6080 | Viruses_unclassified_unclassified_unclassified_unclassified_unclassified_Marine virus AFVG_25M68 | *Streptococcus_oralis* |
|  | SD2_contig_1103 | Viruses_unclassified_unclassified_unclassified_unclassified_unclassified_Prokaryotic dsDNA virus sp. | *Chromobacterium_haemolyticum* |
|  | SD3_contig_14231 | Viruses_unclassified_unclassified_unclassified_unclassified_unclassified_Prokaryotic dsDNA virus sp. | *Lacinutrix_sp._Bg11-31* |
|  | SA1_contig_2678 | Viruses_unclassified_unclassified_unclassified_unclassified_unclassified_Prokaryotic dsDNA virus sp. | *Maritimibacter_alkaliphilus* |
|  | SB3_contig_897 | Viruses_unclassified_unclassified_unclassified_unclassified_unclassified_Prokaryotic dsDNA virus sp. | *Porphyromonas_gingivalis* |
|  | SD1_contig_6867 | Viruses_unclassified_unclassified_unclassified_unclassified_unclassified_Prokaryotic dsDNA virus sp. | *Yersinia_pekkanenii* |

The hosts of the phage were predicted by comparing identified phages with the CRISPR-Cas spacer database.

**Table S8** **Correlation test between the relative abundance of phage and the relative abundance of prokaryote.**

| Sample | phage | prokaryote | R | *p* |
| --- | --- | --- | --- | --- |
| Intestine | Microviridae | *Vibrio* | -0.656 | 0.033 |
|  | Siphoviridae | *Formosa* | 0.617 | 0.037 |
|  | Herelleviridae | *Shewanella* | 0.632 | 0.035 |
| sediment | Podoviridae | *Algoriphagus* | 0.577 | 0.050 |
|  | Podoviridae | *Flavobacterium* | 0.713 | 0.023 |
|  | Podoviridae | *Rheinheimera* | 0.667 | 0.033 |
|  | Siphoviridae | *Formosa* | 0.732 | 0.023 |
|  | Microviridae | *Shewanella* | -0.773 | 0.015 |
|  | Microviridae | *Paracoccus* | 0.645 | 0.034 |
|  | Inoviridae | *Aurantisolimonas* | 0.783 | 0.015 |

Relative abundance of phage (family level) and prokaryote (genus level) pairs with significant correlation, which were dominated in the microbial community in shrimp intestine and sediment, and the Pearson correlation coefficient was indicated.
